# Cross-species genomic and epigenomic landscape of retinoblastoma

**DOI:** 10.18632/oncotarget.1051

**Published:** 2013-05-11

**Authors:** Claudia A. Benavente, Justina D. McEvoy, David Finkelstein, Lei Wei, Guolian Kang, Yong-Dong Wang, Geoffrey Neale, Susan Ragsdale, Virginia Valentine, Armita Bahrami, Jamshid Temirov, Stanley Pounds, Jinghui Zhang, Michael A. Dyer

**Affiliations:** ^1^ Department of Developmental Neurobiology, St. Jude Children's Research Hospital, Memphis, TN, USA; ^2^ Hartwell Center for Bioinformatics and Biotechnology, St. Jude Children's Research Hospital, Memphis, TN, USA; ^3^ Department of Computational Biology, St. Jude Children's Research Hospital, Memphis, TN, USA; ^4^ Department of Biostatistics, St. Jude Children's Research Hospital, Memphis, TN, USA; ^5^ Department of Tumor Cell Biology, St. Jude Children's Research Hospital, Memphis, TN, USA; ^6^ Department of Pathology, St. Jude Children's Research Hospital, Memphis, TN, USA; ^7^ Cell and Tissue Imaging Facility, St Jude Children's Research Hospital, Memphis, Tennessee, USA; ^8^ Howard Hughes Medical Institute, Chevy Chase, MD

**Keywords:** retinoblastoma, RB1, epigenetics

## Abstract

Genetically engineered mouse models (GEMMs) of human cancer are important for advancing our understanding of tumor initiation and progression as well as for testing novel therapeutics. Retinoblastoma is a childhood cancer of the developing retina that initiates with biallelic inactivation of the *RB1* gene. GEMMs faithfully recapitulate the histopathology, molecular, cellular, morphometric, neuroanatomical and neurochemical features of human retinoblastoma. In this study, we analyzed the genomic and epigenomic landscape of murine retinoblastoma and compared them to human retinoblastomas to gain insight into shared mechanisms of tumor progression across species. Similar to human retinoblastoma, mouse tumors have low rates of single nucleotide variations. However, mouse retinoblastomas have higher rates of aneuploidy and regional and focal copy number changes that vary depending on the genetic lesions that initiate tumorigenesis in the developing murine retina. Furthermore, the epigenetic landscape in mouse retinoblastoma was significantly different from human tumors and some pathways that are candidates for molecular targeted therapy for human retinoblastoma such as SYK or MCL1 are not deregulated in GEMMs. Taken together, these data suggest there are important differences between mouse and human retinoblastomas with respect to the mechanism of tumor progression and those differences can have significant implications for translational research to test the efficacy of novel therapies for this devastating childhood cancer.

## INTRODUCTION

Retinoblastoma is a rare childhood cancer of the developing retina that initiates with biallelic loss of the *RB1* gene [1]. *RB1* inactivation confers limitless replicative potential to retinoblasts and these preneoplastic cells can progress to retinoblastoma by acquiring additional cellular properties including evasion of cell death and senescence, sustained angiogenesis and activation of growth-signaling pathways. Several different mechanisms have been proposed to explain the rapid progression of retinoblastoma following *RB1* inactivation. In a series of elegant studies using genetically engineered murine cells and immortalized human cells, it was shown that *RB1* plays an important role in maintaining genomic stability [2-4]. Thus, in some cellular contexts, inactivation of the *RB1* gene could lead to chromosome instability (CIN), allowing secondary and tertiary mutations in key cancer pathways to be rapidly acquired. Alternatively, *RB1* has also been implicated in a variety of epigenetic processes (reviewed by [5]) so it is also possible that perturbations in the epigenetic landscape may contribute to tumorigenesis in the retina. In support of an epigenetic mechanism, recent whole-genome sequencing and integrated epigenetic analysis of human retinoblastoma revealed that the tumors have relatively stable genomes and several cancer genes were epigenetically deregulated. At least one of those epigenetically deregulated genes (*SYK*) is required for retinoblastoma tumor cell survival in vivo [6]. These two alternative mechanisms (genome instability and epigenetic deregulation) of retinoblastoma progression are not necessarily mutually exclusive and some tumors may show evidence of both chromosomal instability and epigenetic deregulation.

Over the past 8 years, a series of knockout mouse models of retinoblastoma have been generated by conditionally inactivating multiple Rb family members in the developing retina [7-9]. Knockout mouse models of retinoblastoma have been valuable for studying the contribution of other tumor suppressor pathways such as the p53 pathway [8] and for testing novel therapeutic agents for the treatment of retinoblastoma [10, 11]. In one study, 6 different strains of mice that develop retinoblastoma were analyzed side-by-side using the same retinal progenitor specific Cre transgene (*Chx10-Cre*)[9]. Histopathological analysis, gene expression profiling, morphometric, neuroanatomical and neurochemical analyses [9] showed that mouse retinoblastomas faithfully recapitulate the molecular and cellular features of human retinoblastomas. However, while the timing of retinoblastoma initiation was indistinguishable across the 6 strains, tumor penetrance and the rate of progression varied dramatically [9]. These data raise the possibility that the genetic/epigenetic changes that accompany human retinoblastoma progression may not be faithfully recapitulated in the mouse despite the remarkable inter-species similarities at the molecular and cellular level. This could have important implications for interpreting preclinical testing of novel molecular targeted therapeutics in murine models of retinoblastoma. In fact, preclinical testing of combination chemotherapy (etoposide, carboplatin and vincristine) showed dramatic differences in response between genetically engineered mouse models (GEMMs) and human orthotopic xenografts; virtually all of the GEMMs were cured of their disease while there was no improvement in progression free survival or overall survival in the orthotopic xenograft model [12]. It is possible that such species-specific differences could be further amplified when testing molecular targeted therapeutics directed toward processes important for maintaining genomic stability or the epigenetic landscape of retinoblastoma.

In this study, we characterized the genomic and epigenomic landscape of murine retinoblastoma and provide a direct comparison to human retinoblastoma. Genome stability was measured by ploidy and gross chromosomal rearrangements (GCR). To explore the underlying mechanisms, we measured sister chromatid cohesion, kinetics of double strand DNA (dsDNA) damage repair, oxidative stress and expression of proteins implicated in maintenance of genome stability across species. In order to identify any possible secondary or tertiary genetic lesions in mouse retinoblastomas, we also performed array comparative genome hybridization (aCGH) and exome sequencing. Finally, to characterize the epigenetic landscape in mouse retinoblastoma we performed integrated analysis using gene expression data, DNA methylation data and analysis of histone marks associated with active or silent chromatin. Taken together, these data suggest that even though the overall molecular and cellular features of mouse and human retinoblastomas are remarkably similar, there are critical differences in the genomic and epigenomic landscapes across species. This may suggest that not only are the effects of *RB1* inactivation cell-context specific but they may also be species specific. These data have important implications for our understanding of the role of *RB1* in tumorigenesis, for modeling human cancer in the mouse and for interpreting preclinical data using GEMMs and human orthotopic xenografts.

## RESULTS

### Conserved Gene Expression Signature in Mouse and Human Retinoblastoma

**Figure 1:**
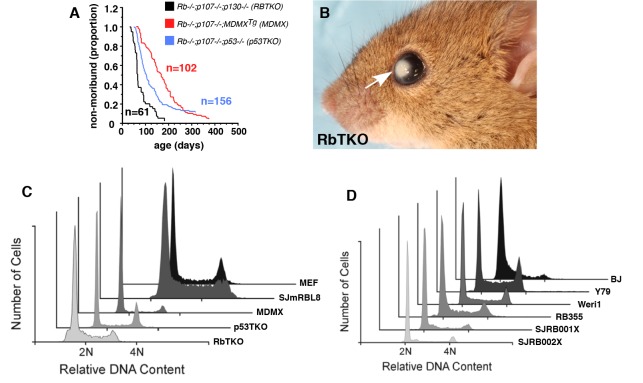
Analysis of DNA Content in Mouse and Human Retinoblastoma (A) Survival curves for the three strains of mice used in this study. (B) Representative image of a late stage tumor from an RbTKO mouse showing anterior chamber invasion (arrow). (C) Quantitative analysis of DNA content for mouse retinoblastoma tumors taken directly from the mice (Rb TKO, p53 TKO and MDMX) as well as a mouse retinoblastoma cell line (SJmRBL8 and a control wild type MEF (wt)). (D) Quantitative analysis of DNA content for human retinoblastoma cell lines (Y79, Weri1, RB355), two orthotopic xenografts (SJRB001X and SJRB002X) and Tert immortalized fibroblasts as a normal diploid control (BJ).

We compared primary mouse tumors from three different strains (Fig. 1A-B) to human retinoblastoma to establish if any of these GEMMs recapitulates human retinoblastoma gene expression more closely. RbTKO mice (*Chx10-Cre;Rb*^*Lox/Lox*^*;p107*^*−/−*^*;p130*^*Lox/Lox*^) have mosaic conditional inactivation of all 3 Rb family members in retinal progenitor cells during development [9]. p53TKO mice (*Chx10-Cre;Rb*^*Lox/Lox*^*;p107*^*−/−*^*;p53*^*Lox/Lox*^) combine Rb pathway inactivation with p53 pathway inactivation [7-9]. While the *TP53* gene is not mutated in human retinoblastoma [8], these mice serve as a convenient positive control for our studies because p53 gene inactivation can lead to defects in DNA repair and contribute to genome instability [13, 14]. *MDMX* mice (*Chx10-Cre;Rb*^*Lox/Lox*^*;p107*^*−/−*^*;MDMX*^*Tg*^) have conditional overexpression of the *MDMX* gene to mimic the elevated expression of *MDMX* (Mdm4) in human retinoblastomas [8]. To determine the statistical significance of the similarity between the gene expression profiles of mouse and human retinoblastomas, we ran the agreement of differential expression (AGDEX) analysis using tumor and normal retina gene expression array data [15]. The differential expression statistics for the mouse comparison and human comparison for each of the 79,361 probe-set pairs for RbTKO, p53TKO and *MDMX* model is plotted in Supplemental Figure 1. We also computed the agreement statistics and *P-*values for each gene-set. A respective totals of 66, 16 and 20 gene-sets for RbTKO, p53TKO and *MDMX* models had the robust result of showing significance at the *P* = 0.01 level in each of the four permutation tests performed by computing each of two agreement statistics across permutations of group labels for each of two agreement statistics (Sup. Table 1). Overall, 68.8% of 13,823 ortholog probe pairs in RbTKO, 71.2% of 11,273 ortholog probe pairs in *MDMX*, and 69.6% of 11,059 ortholog probe pairs in p53TKO showed agreement in gene expression (upregulation or downregulation) between human retinoblastoma and the respective mouse counterpart (Sup. Table 1). These data are consistent with previous molecular, cellular, histopathological, electron microscopic, morphometric and neuroanatomical studies showing that the RbTKO, *MDMX* and p53TKO mouse retinoblastomas are indistinguishable [9]. The agreement of differential expression observed for the three genetic mouse models compared to human retinoblastoma, is higher than what was previously reported for genetic mouse models of medulloblastoma considered to be a benchmark of GEMMs in pediatric cancer [16].

### Analysis of DNA Content and Ploidy in Human and Mouse Retinoblastoma

As a first step toward determining if mouse retinoblastomas have defects in chromosome segregation that may lead to aneuploidy and change in DNA content, we performed FACS analysis of DNA content of mouse and human retinoblastoma cell lines, primary tumors and orthotopic xenografts. We used mouse embryonic fibroblasts (MEFs) as normal diploid control for mouse retinoblastoma and TERT-immortalized human fibroblasts (BJ) as a diploid control for human retinoblastoma [17]. All analyses were performed side-by-side to obtain quantitative comparisons across samples. Primary retinoblastomas from each of the GEMMs strains showed changes in overall DNA content as compared to the diploid control (Fig. 1C). A previously developed mouse retinoblastoma cell line (SJmRBL8) [18] also showed evidence of changes in DNA content consistent with aneuploidy (Fig. 1C). In contrast to these results from mouse retinoblastomas and cell lines, human retinoblastoma cell lines [19] and orthotopic xenografts [9] showed no deviation from the normal distribution in DNA content as compared to the diploid control (Fig. 1D). These data are consistent with the previously published data showing that most retinoblastomas have a diploid or near-diploid karyotype [20].

To extend and validate the results from the FACS analysis, we performed spectral karyotype analysis (SKY) on primary tumors from each of the 3 mouse strains and both human orthotopic xenografts (Sup. Fig. 2, Tables 1, 2). Overall, the mouse retinoblastomas exhibited aneuploidy but had no evidence of other types of GCRs (i.e. translocations, isochromosomes, and double minute formation) based on the SKY analysis (Sup. Fig. 2, Table 1). Interestingly, there was a significantly broader distribution of chromosome number in the p53TKO retinoblastoma tumors than MDMX and RbTKO retinoblastomas (Table 1). Moreover, statistical analysis of the SKY data showed that the RbTKO retinoblastomas had the highest probability of retaining a normal karyotype (retention probability=0.4773) (Sup. Table 2). In contrast, aneuploidy was less prevalent in the human retinoblastoma samples (retention probability=0.75) but there was evidence of other types of GCRs (Sup. Fig. 2, Table 2, Sup. Table 2).

**Table 1:** Results from SKY analysis for mouse retinoblastoma

Sample	Cells scored	^1^Chr count	^2^del (%)	^3^gains (%)	^4^total aneuploidy (%)
p53TKO	23	36(1), 39(2), 40(8), 41(1), 44(1), 45(1), 60(1), 71(1), 72(1), 78(1), 80(2), 83(1), 84(1), 100(1)	43.5	47.8	65.2*
MDMX	13	43(6), 44(7)	0	100	100*
RbTKO	24	38(2), 39(1), 40(20), 42(1)	16.7	8.3	20.8

1 Chromosomal count per cell, the number in parenthesis represents the number of cells that present the indicated chromosomal event

2 del=percentage of cells with chromosomal deletions

3 gains=percentage of cells with chromosomal gains

4 percentage of cells presenting aneuploidy (chromosomal gains or deletions)

* p>0.001 compared to RbTKO

**Table 2:** SKY Analysis for Human Retinoblastoma Xenografts

^1^Sample	Cells scored	^2^Chr count	^3^gains (%)	^4^del (%)	^5^Total aneuploidy (%)	^6^i(6)(p10) (%)	^7^der (%)	^8^dmin (%)	^9^dup (%)	^10^t (%)
SJRB001X	24	44(1), 45(2), 46(3), 47(18)	0	25	25	100	8.3	83.3	0	0
SJRB002X (2R.1)	15	47(15)	0	0	0	100	100	0	0	0
SJRB002X (4R.1)	8	46(1), 47(7)	0	0	0	87.5	100	0	12.5	0
SJRB002X (80R.1)	13	45(3), 46(10)	0	100	100	100	100	0	0	0
SJRB002X (2R.2)	15	47(15)	0	0	0	100	100	0	0	0
SJRB002X (4R.2)	14	45(5), 46(1), 47(8)	0	42.9	42.9	100	100	0	0	7.1
SJRB002X (80R.2)	15	46(13), 47(2)	0	87	87	100	100	0	0	0

1 The designations in parentheses indicate individual sublines (2R, 4R, 80R) and passage (2R.1 is passage 1 and 2R.2 is passage 2).

2 Chromosomal count per cell, the number in parenthesis represents the number of cells that present the indicated chromosomal event

3 gains=percentage of cells with chromosomal gains

4 del=percentage of cells with chromosomal deletions

5 percentage of cells presenting aneuploidy (whole chromosome gains or deletions)

6 i=percentage of cells with isochromosome

7 der=percentage of cells with derivative chromosomes

8 dmin=percentage of cells with double minutes

9 dup=percentage of cells with duplication

10 t=percentage of cells with translocations

DNA content analysis and SKY provide quantitative data on chromosomal content and integrity at a single timepoint in individual cells but cannot distinguish between an acute event that leads to changes in chromosomal ploidy or stability [21] versus continuous defects in chromosome segregation. To test this directly, we analyzed clones of cells derived from individual mouse and human retinoblastoma cells. Primary human and mouse retinoblastomas do not grow from single cells in culture so we used the Y79 human retinoblastoma cell line (Sup. Fig. 3A,B) and the SJmRBL8 mouse retinoblastoma cell line (Sup. Fig. 3C,D) [18, 22]. Single cells were seeded into individual wells of 96 well dishes and 3 individual clones for each cell line were expanded. We karyotyped 20 cells for each clone and the parental cell lines and scored the number of chromosomal gains and losses (Sup. Fig. 3E). We found that there was more intra-clonal heterogeneity in the mouse retinoblastoma cell line clones than in the human retinoblastoma cell line clones (Sup. Fig. 3E).

### Analysis of Sister Chromatid Cohesion in Mouse Retinoblastomas

**Figure 2:**
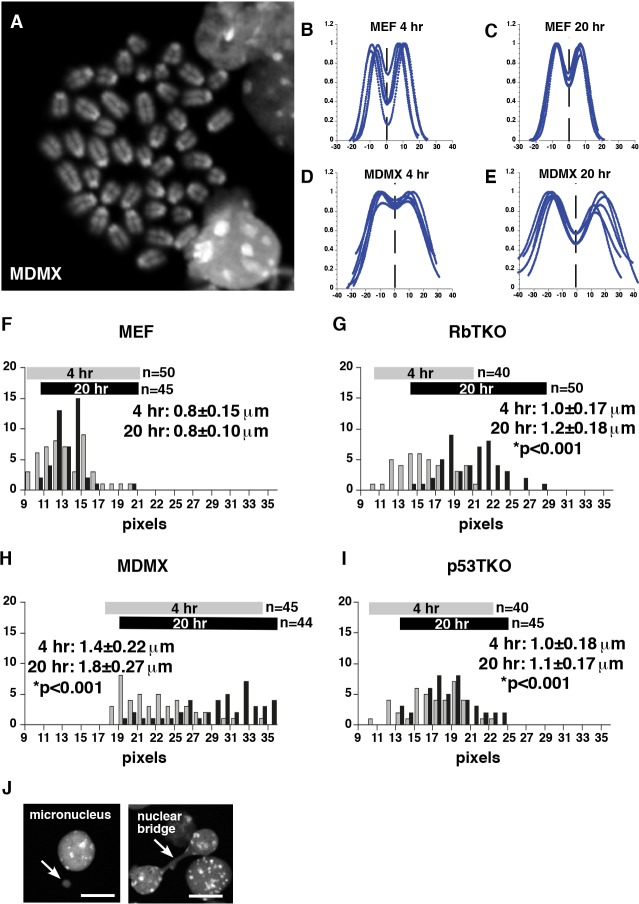
Analysis of Sister Chromatid Cohesion in Mouse Retinoblastoma (A) Representative image of DAPI-stained chromosome spread from a retinoblastoma in the MDMX mouse strain (*Chx10-Cre;Rb*^*Lox/Lox*^*;p107*^*−/−*^*;MDMX*^*Tg*^). (B–E) Representative tracing showing the relative fluorescence intensity across 5 pairs of sister chromatids for mouse diploid fibroblasts (MEF) and primary mouse MDMX retinoblastoma cells after 4-hr or 20-hr colcemid treatments. Distance between the peaks is plotted in pixels. (F-I) Histogram of the number of chromosomes with the indicated sister chromatid distance is plotted for MEF, RBTKO, MDMX and p53TKO samples after 4-hr or 20-hr colcemid treatments. The mean and standard deviation were calculated from the indicated number of measurements from 3 independent experiments. * p values relative to MEF controls. (J) Examples of micronuclei and nuclear bridges in p53TKO mouse retinoblastoma. Scale bar: 5μm.

Several studies in mice and cultured cells have linked *RB1* inactivation to defects in chromosome segregation that can contribute to tumor cell aneuploidy [2, 3, 23-25] and CIN [2]. Specifically, in cultured human RPE cells, *RB1* knockdown results in a defect in sister chromatid cohesion that makes the chromatids more prone to separate when cells are delayed in mitosis [2]. Defects in sister chromatid cohesion can lead to aneuploidy and may, in turn, contribute to CIN by forming merotelic kinetochore attachments [26-28]. Previous studies have shown that human *RB1*-deficient retinoblastoma cells have defects in sister chromatid cohesion [6] consistent with previously published data on cultured human RPE cells with *RB1* depletion [2]. To determine if changes in sister chromatid cohesion correlate with increased aneuploidy in mouse retinoblastoma cells, we analyzed the distance between sister chromatids in colcemid-treated mouse retinoblastoma tumor cells from RbTKO, MDMX and p53TKO strains and the SJmRBL8 mouse retinoblastoma cell line. The mouse retinoblastoma cells had a significant increase in sister chromatid cohesion in all mouse retinoblastoma cells compared to MEF cells after 4 hr and 20 hr of colcemid treatment (*p*<0.01) (Fig. 2A–I and data not shown). In addition, there was a subtle increase in micronuclei and nuclear bridges in some of the retinoblastoma samples (Fig. 2J and Sup. Table 3). Overall, these data are consistent with previously published data on human retinoblastomas and *RB1*-depleted human RPE cells showing that loss of *RB1* leads to defects in sister chromatid cohesion [2].

### Analysis of Chromosomal Lesions and Double Strand–Break Repair in Retinoblastoma

**Figure 3:**
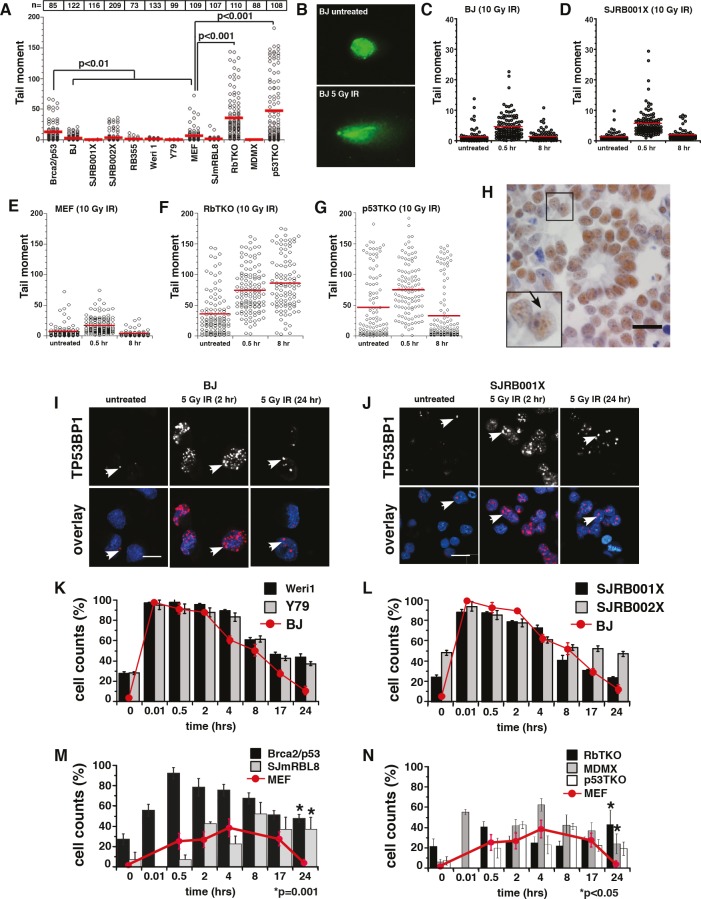
Analysis of dsDNA-Break Repair in Retinoblastoma (A) Comet analysis of retinoblastoma cell lines, human retinoblastoma xenografts, and primary mouse retinoblastoma tumors. The number of cells analyzed in each experiment is indicated at the top of the plot. TERT-immortalized human fibroblasts (BJ) were used as the normal human negative-control; MEF cells were used as the normal mouse negative-control, and *Brca;p53*-deficient medulloblastoma cells were used as the positive control. (B-G) Cells were exposed to 10 Gy irradiation (IR), and dsDNA-break repair of BJ and SJRB001X tumor cells (C, D), MEF, RbTKO and p53TKO tumor cells (E-G) was monitored by performing comet assays at different time points. (B) Representative images of nuclei stained with Sytox green before and after exposure to 10 Gy IR. (H) Immunostaining of primary human retinoblastoma to detect TP53BP1 foci (arrow). (I, J) Immunofluorescence detection of TP53BP1 (red) in DAPI-stained nuclei (blue) of untreated BJ and SJRB001X cells and after exposure to 5 Gy IR. Foci of TP53BP1 at sites of dsDNA breaks are highlighted with arrows. Histogram of the proportion of cells with TP53BP1 foci before and after exposure to 5 Gy IR in (K) human retinoblastoma cell lines (Weri1 and Y79), (L) two independent retinoblastoma orthotopic xenografts (SJRB001X and SJRB002X), (M) mouse retinoblastoma cell line (SJmRBL8), and (N) the three primary mouse retinoblastoma (RBTKO, MDMX, p53TKO). p values compared to untreated samples.

Tumor cells with high rates of dsDNA breaks or defects in dsDNA-break repair may accumulate GCRs such as translocations, inversions, double minutes and isochromosomes [29-31]. To test the integrity of the retinoblastoma genome directly, we performed comet assays on human and mouse retinoblastoma cell lines, mouse retinoblastoma tumors, and human retinoblastoma orthotopic xenografts. BJ (human) and MEF (mouse) cells were used as normal controls, and *Brca2;p53* deficient murine medulloblastoma cells were used as a positive control because these cells have defects in dsDNA-break repair [32]. The DNA fragmentation of the *Brca2;p53*-deficient cells was significantly increased compared to the wild type control cells (*p*<0.001, Fig. 3A). Human retinoblastoma cells as well as the mouse cell line SJmRBL8 and *MDMX* tumor cells were indistinguishable from the wild type control cells in this assay, but mouse RbTKO and p53TKO retinoblastoma cells had significantly higher basal levels of DNA breaks compared to the MEF control (*p*<0.001, Fig. 3A).

Next, we induced sub-lethal levels of dsDNA breaks by exposing the cells to 10 Gy of ionizing radiation (IR), the minimum dose at which all cells showed a significant increase in dsDNA breaks using the comet assay. We monitored the integrity of the genome 30 minutes, 8 hours or 24 hours after exposure to 10 Gy IR (Fig. 3B-G and data not shown). We found that control cells, human retinoblastoma cells and mouse *MDMX* and p53TKO retinoblastomas repaired DNA breaks within 8 hours, while RbTKO retinoblastomas failed to repair DNA damage even after 24 hours (*p*<0.001, Fig. 3C-G and data not shown).

**Figure 4:**
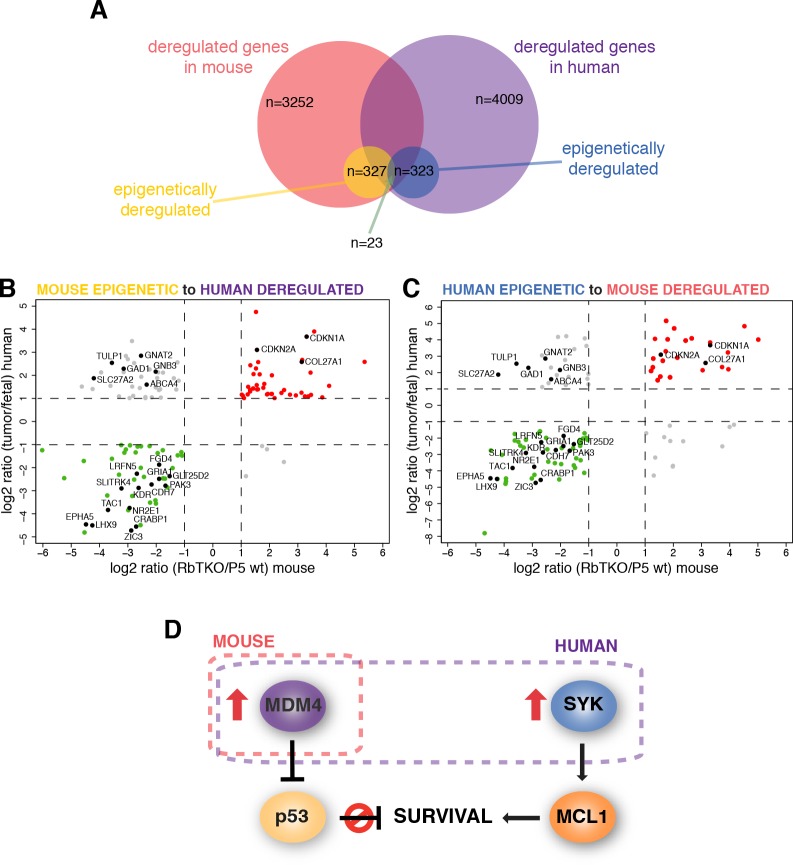
Mouse and Human Integrative Data Analysis for Retinoblastoma Integrative data analysis for mouse retinoblastoma was compared to the human retinoblastoma integrative data from Zhang et al[6] (A) Diagram showing the number of genes significantly deregulated in mouse and human retinoblastoma, the subset considered epigenetically deregulated by our integrative data analysis in each specie and those epigenetically deregulated both in mouse and human. (B) Gene expression data of the 131 genes epigenetically deregulated in the RbTKO mouse retinoblastoma model that could be matched to genes in the human retinoblastoma integrative data. (C) Gene expression data of the 111 genes epigenetically deregulated in human retinoblastoma that could be matched to genes in the RbTKO mouse retinoblastoma. Genes up- and down-regulated in both mouse and human are depicted in red and green, respectively. Genes deregulated in opposite directions are colored in gray. Genes found to be epigenetically deregulated in both species are labeled (black dots). (D) Proposed survival pathways for mouse and human retinoblastoma.

To complement the comet assays, we analyzed the focal accumulation and resolution of proteins that target dsDNA breaks (γ-H2AX and TP53BP1) in human and mouse retinoblastoma cells. First, we used a tissue microarray to analyze the expression pattern of TP53BP1 on 90 primary human retinoblastomas (Fig. 3H) and found that a subset of primary human retinoblastomas had elevated endogenous levels of TP53BP1 nuclear foci (Sup. Table 4 and Fig. 4H) although the expression of TP53BP1 and the number of chromosomal lesions detected was not correlated (data not shown). TP53BP1 expression on 3 of each of the mouse retinoblastoma models was also analyzed and we found extensive endogenous levels of TP53BP1 nuclear foci in 100% of the samples analyzed (data not shown).

Next, we analyzed the resolution of dsDNA breaks (γ-H2AX and TP53BP1 foci) in human and mouse retinoblastoma cells following exposure to 5 Gy IR. We used a lower dose of IR for these experiments than the previous comet assays in order to resolve the individual immunofluorescent γ-H2AX and TP53BP1 foci on confocal micrographs. Retinoblastoma cell lines, mouse tumors and the human orthotopic xenografts had slightly more basal γ-H2AX and TP53BP1 foci than did normal diploid control cells (Fig. 3I-N and data not shown). Treatment with 5 Gy IR led to an increase in the number of foci per cell, and these foci were largely resolved by 24 hours for the human retinoblastomas (Fig. 3I-L). The kinetics of resolution of the dsDNA-break repair in human was similar to that of control cells. However, all mouse retinoblastoma cells displayed a significant reduction in their ability to resolve TP53BP1 foci over 24 hours, though p53TKO doesn't reach statistical significance (*p*=0.2, Fig. 3M,N).

Another source of stress that may contribute to aneuploidy and CIN in mouse retinoblastomas is oxidative stress [33]. Indeed, the spectrum of SNVs in human retinoblastoma whole genome sequence data are consistent with elevated levels of reactive oxygen species in the tumor cells [34]. To determine whether retinoblastomas have elevated levels of oxidative stress, we quantified reactive oxygen species by using the oxidation-sensitive dye DCF-DA in flow cytometry experiments (Sup. Fig. 4). We found that mouse retinoblastoma cells had evidence of oxidative stress using this assay (Sup. Fig. 4 and data not shown). Interestingly, when we measured the mouse and human retinoblastomas' sensitivity to oxidative stress (i.e., H2O2 exposure) compared to that of our normal control cells (BJ and MEF), we observed a decrease in DCF-DA oxidation, except for mouse RbTKO (Sup. Fig. 4). This decrease in DCF-DA oxidation correlated with a significant increase in cell death in tumors and could be reversed by treatment with antioxidants or catalase (Sup. Fig.4 and data not shown). These data suggest that some retinoblastomas are at their maximum tolerable oxidative stress levels (except RbTKO) and that this may contribute to elevated levels of dsDNA breaks and G-to-T or C-to-A transversions [6].

### Analysis of Genetic Changes in Mouse Retinoblastoma

Whole genome sequence analyses of human retinoblastoma showed a low rate of aneuploidy, structural variations (SVs) and copy number variations compared to tumors with chromosomal instability [6]. To determine if this low rate of chromosomal lesions is also found in mouse retinoblastomas and if the defects in dsDNA break repair contribute to increased rates of chromosomal lesions in the RbTKO mouse retinoblastomas, we performed aCGH analyses for 6 tumors of each of the mouse retinoblastoma models (Sup. Fig. 5). Gains or losses of whole chromosomes were observed in approximately half of the tumors from each genotype (Sup. Table 5). In agreement with our previous SKY analysis, p53TKO tumors had an average of 3.83 chromosomal gains/losses compared to 0.67 for both RbTKO and MDMX tumors (p<0.1). A maximum of 10 chromosomal gains/losses were present in one of the p53TKO tumors (P53-169). The most common recurrent whole chromosome lesion was a gain of chromosome 12 across each of the mouse models (3/6 RbTKO, 2/6 MDMX, and 3/6 p53TKO) (Sup. Table 5). Chromosome 12 contains a region of synteny with human 14q, a chromosome including 14q32, a region for which translocations has been previously described in human retinoblastoma [20]. No copy number gains or epigenetic changes have been reported for genes in this region in human retinoblastoma.

In addition to whole chromosome gains and losses that are characteristic of aneuploidy, there were also several large recurrent chromosomal regions (≥3Mb) in a subset of tumors among RbTKO, MDMX and p53TKO (Sup. Table 6). The overall average number of gains or losses of large chromosomal regions (≥3Mb) was 1.83 for RbTKO, 1.00 per MDMX and 0.50 per p53TKO and the maximum was 7 in a single tumor (TKO-841) (Sup. Table 6). In all tumor models, 66.7% (4/6) had no regional chromosomal gains or losses (Sup. Table 6). Of interest, we observed recurrent regional chromosomal lesions in chromosome 12 in regions of synteny to human chromosome 14q32 in 3/18 samples. In total, 55% (10/18) of mouse retinoblastomas had whole or regional chromosomal gains on chromosome 12.

A striking difference was observed in the number of focal lesions (20kb- 3Mb) in the RbTKO compared to MDMX and p53TKO (p<0.01). On average, the RbTKO tumors had 176.2 focal lesions per tumor compared to 19.3 per MDMX and 22.1 per p53TKO. In the RbTKO tumors, there were a total of 43 recurrent focal lesions in at least 4/6 tumor samples (Sup. Table 7). Within the recurrent focal gains or losses in the RbTKO retinoblastoma model, we found 12 in cancer genes, including *Lmo1* in 5/6 samples; *Ndrg1*, *Brd4*, *Fbxo11*, and *Rbm15* in 4/6 samples; *Mdm4*, *Msi2*, and *Ezh2* in 3/6 samples. Overall, there is a single recurrent focal chromosomal amplification among all three mouse retinoblastoma models, in the Nlrp1b, a member of the Ced-4 family of apoptosis proteins.

To identify single nucleotide variations (SNVs) and indels in the RbTKO tumors, we also performed whole exome sequencing of 11 tumors. Single nucleotide variations (SNV) were found in 63.6% (7/11) (Sup. Table 8) and indels were found in 54.5% (6/11) of the tumors (Sup. Table 9). All SNVs and indels were validated by custom capture and Illumina sequencing. On average there were 3.2 somatic mutations (SNV and indels) per tumor in the RbTKO mouse model. There were no recurrently mutated genes in this cohort nor were any known cancer genes mutated in the 11 tumors.

### Integrated Epigenetic Analysis of Mouse Retinoblastoma

Analyses of human retinoblastoma genome and epigenome uncovered few genetic lesions and identified epigenetic as the mechanism underlying the rapid progression of human retinoblastoma [6]. To determine if there were any epigenetically deregulated genes in mouse retinoblastoma that were shared with human retinoblastoma, we performed DNA methylation analysis and chromatin immunoprecipitation (ChIP) assays for histone marks of actively transcribed (H3K4me3 and H3K9/14Ac) or silent chromatin (H3K9me3). We integrated data on gene expression, DNA methylation and histone marks for RbTKO tumors and P5 mouse retina to be able to compare our data to the previously published data on integrated epigenetic analysis of human retinoblastoma and human fetal retina. Overall, we found that the epigenetic landscape in mouse retinoblastoma was very different than for human retinoblastoma, with 327 genes being epigenetically deregulated (Figure 4A and Sup. Table 10). Among the 327 epigenetically regulated genes in mouse retinoblastoma, only 23 were also epigenetically deregulated in human retinoblastoma, 17 of which change gene expression in the same direction (Figure 4B,C). In the mouse retinoblastoma, 11 of the epigenetically deregulated genes are cancer genes but none of those were found to be epigenetically deregulated in the human retinoblastomas (Sup. Table 10).

**Figure 5:**
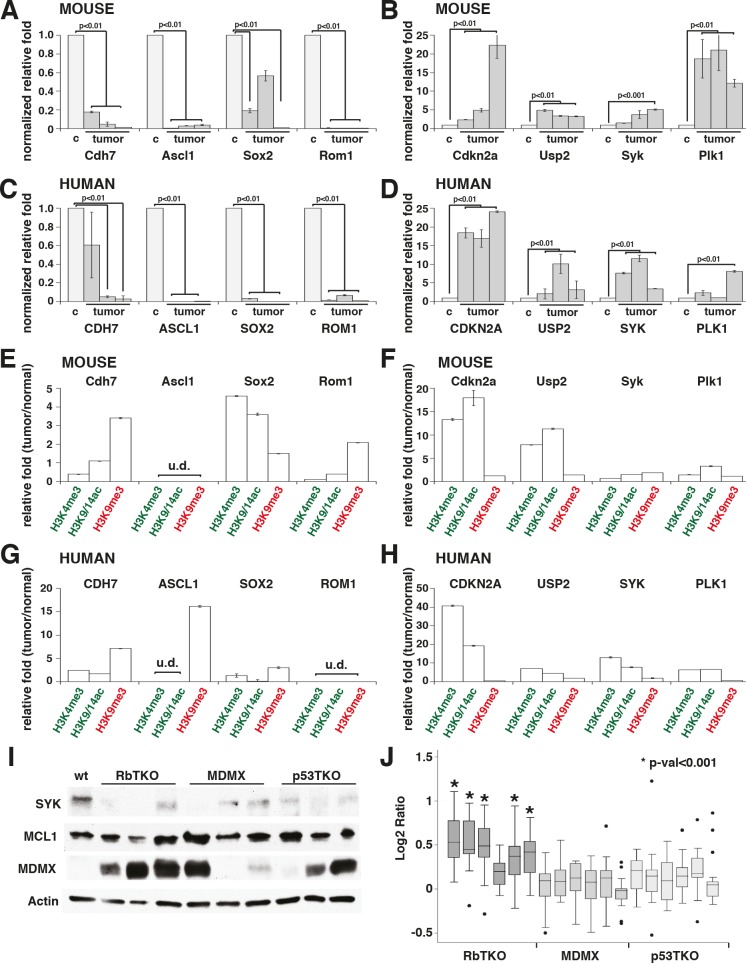
SYK Is Not Epigenetically Deregulated in Mouse Retinoblastoma (A-D) Gene expression validation for a subset of genes downregulated (A, C) and upregulated (B, D) in mouse RbTKO tumors (A-B) and human retinoblastoma (C-D). In (A-B), control (c) is postnatal day 5 wilt type (P5 wt) mouse retina and tumor are three independent RbTKO retinoblastoma tumors. In (C-D), control is fetal week 20 normal retina and tumor are three independent primary human retinoblastomas. Data are presented as the mean and standard deviation of duplicate samples, and all are normalized to GPI1 expression relative to that of control retina. (E–H) ChIP validation for histone marks at the promoter region in mouse (E-F) and human (G-H) tumors using non-amplified ChIP DNA. Green indicates activating histone marks and red indicates repressive histone marks. Each bar is the mean and standard deviation of triplicate samples. (I) Immunoblot of SYK, MCL1, MDMX and actin from retinoblastoma cell lines and P5 wt control retinae. (J) Array-comparative genomic hybridization (aCGH) data for the MDMX gene in RBTKO, MDMX and P53TKO tumor samples.

The changes in gene expression and histone marks for a subset of genes were validated by real time RT-PCR, including some of the cancer genes epigenetically deregulated in human retinoblastoma (*SYK*, *ASCL1* and *SOX2*, Fig. 5A-D and data not shown). Interestingly, we found that *SYK*, a gene that is epigenetically upregulated in human retinoblastoma and is required for retinoblastoma survival, is not epigenetically deregulated in mouse retinoblastoma (Fig. 5B, F and J). Furthermore, the pathway through which it exerts its anti-apoptotic properties does not appear to be deregulated, as MCL1 levels are comparable to P5 wt retinae (Fig. 5I). However, RbTKO mouse retinoblastomas may avoid apoptosis through the amplification of the *MDMX* gene and increased expression of MDMX protein suggesting a common mechanism of p53 pathway inactivation across species (Figure 4D and Fig. 5I-J).

## DISCUSSION

In this study, we analyzed the species-specific role of RB1 in maintaining genome stability and the epigenetic landscape in the developing retina as it relates to retinoblastoma tumor initiation and progression. Like the human retinoblastomas, there were no recurrent somatic SNVs or indels in whole exome sequence analysis of murine retinoblastoma. However, in contrast to human retinoblastoma, we found that mouse retinoblastomas from three different GEMMs had evidence of aneuploidy and chromosomal instability. We also performed the first integrated epigenetic analysis of a mouse tumor model (RbTKO) and compared the epigenome of mouse retinoblastoma to human retinoblastoma. We found that there were a significant number of epigenetically deregulated genes in mouse retinoblastomas and some of those were cancer genes but there was very little overlap with the human epigenome of retinoblastoma. Thus, these data suggest that there are differences in the relative contribution of chromosomal and epigenetic changes in human and mouse retinoblastoma despite robust conservation at the molecular and cellular level. There are important implications of these data for preclinical testing of new therapeutics for the treatment of retinoblastoma and this comparison may be relevant to other GEMMs of human cancer. This is especially true in cases where genetic lesions such as p53 mutations are engineered into GEMMs to promote tumor penetrance and/or progression even when the pathway is not mutated in the corresponding human tumors.

### Cross-Species Comparison of Retinoblastoma

Retinoblastoma is a relatively simple tumor that initiates with a common genetic lesion (*RB1* inactivation) and progresses rapidly in children. The human *RB1* gene can replace the mouse gene in vivo [35] and most functions of *RB1* are conserved across species. Moreover, many features of retinal development are also conserved across species making retinoblastoma an ideal tumor for cross-species comparison.

Previous studies on GEMMs of retinoblastoma have shown that there is remarkable conservation of gene expression signatures, histopathological features, neuroanatomical, neurochemical and morphological hallmarks across different mouse models of retinoblastoma with a variety of initiating genetic lesions. Importantly, all the tumors initiate during retinal development but progress at different rates. It is not known if these different rates are related to the mechanism of progression, the initiating genetic lesions or a combination of the two.

Array CGH, karyotype analysis, SNP6.0 analysis and whole genome sequencing analysis have shown that a significant proportion of human retinoblastomas have near diploid genomes with few genetic lesions [6]. The *RB1* and *BCOR* (13%) genes are the only genes found to be recurrently mutated in human retinoblastomas to date. The *MYCN* gene is the only known oncogene found to be focally recurrently amplified in human retinoblastomas in about 10% of cases [6, 36, 37]. The functional significance of *BCOR* mutations or *MYCN* amplifications is yet to be established *in vivo* in human retinoblastoma. In contrast to the genetic landscape, integrated epigenetic analysis showed that multiple cancer pathways were epigenetically deregulated in human retinoblastoma. For example, the *SYK* gene was found to be epigenetically upregulated in virtually all human retinoblastomas and it is required for tumor cell survival [6]. These data suggest that while genetic changes may play a role in some retinoblastomas, the majority of tumors progress rapidly following *RB1* inactivation as a result of epigenetic changes. Importantly, aneuploidy and GCRs are not a universal hallmark of human retinoblastomas and are not required for tumorigenesis.

Both human and mouse retinoblastomas had low rates of somatic SNVs or indels in genes. The average number of tier 1 mutations in human retinoblastoma was 4 and the average number of similar mutations in RbTKO was 3.2. However, in contrast to human retinoblastomas, the 3 retinoblastoma GEMMs analyzed in this study showed evidence of aneuploidy and GCRs. Indeed, in the RbTKO strain the average number of focal CNVs was 176 per tumor. Several of these lesions were recurrent such as gains in *Lmo1* in 5/6 RbTKO tumors and *Mdm4* gains in 3/6 tumors. *Lmo1* has been implicated as an oncogene in human neuroblastoma and may contribute to tumorigenesis in this mouse model. However, *LMO1* is not altered in human retinoblastoma at the genomic, epigenomic or transcriptional level. There was no evidence of genetic lesions in *Bcor* or *Mycn* in our mouse model of retinoblastoma making *MDMX* the only common genetic lesion across species in our study.

To determine if there was cross-species conservation of epigenetic processes, we also performed integrated epigenetic analysis of the RbTKO mouse tumors and compared those data to the human retinoblastoma epigenetic landscape. From the integrated analysis, 4009 genes are significantly deregulated in human retinoblastoma (2567 up*-* and 1442 down-regulated) and 3252 in mouse RbTKO tumors (1634 up*-* and 1618 down-regulated). Of these, 8% (323) of the human and 10% (327) of the mouse genes had epigenetic signatures that correlated with the directional changes in gene expression. Gene matching across species, 974 genes were significantly deregulated in both human and mouse retinoblastomas, with 682 (70%) genes changing in the same direction (420 up*-*regulated and 262 down-regulated). Among these 682 genes significantly deregulated in both species, 131 genes of the 327 mouse and 111 of the 323 human genes are epigenetically deregulated. However, only 23 are epigenetically deregulated in both species (17 in the same direction, 2.5%), indicating that while the gene expression changes that occur in mouse and human retinal tumorigenesis may be conserved, the mechanism by which this occurs is not.

### Mechanisms of Retinoblastoma Progression in Murine Retinoblastoma

It has been proposed from previous studies on mouse and human cells that perturbations in the Rb pathway can lead to defects in sister chromatid cohesion and this may contribute to aneuploidy and CIN. We found that human and mouse retinoblastoma cells have defects in sister chromatid cohesion but this does not correlate with aneuploidy or CIN across species or among the different GEMMs analyzed here. We hypothesize that retinoblastomas are predisposed to exhibit CIN because of the absence of *RB1* but this is more likely to occur when additional stress, such as oxidative or replicative stress, overwhelms the cellular machinery that maintain genome stability. It is also possible that effects of *RB1* inactivation on CIN and aneuploidy is cell-type specific and that human retinal cells lacking *RB1* that have aneuploidy or CIN die during tumorigenesis but murine cells are more tolerant of such chromosomal defects [38].

One of the most striking results from our study related to the mechanism of tumor progression was the observation that the RbTKO strain had on average 176 focal CNVs per tumor. Those tumors also had evidence of oxidative stress, elevated levels of DNA breaks and defects in their ability to repair those breaks. While human tumors also have evidence of oxidative stress, they do not result in such elevated levels of CNVs. The same was true for the p53TKO and MDMX GEMMs. While all 3 strains sustain high levels of dsDNA breaks as a result of oxidative stress, the inability of p53TKO and MDMX tumor cells to efficiently repair those breaks makes due to defects in the p53 pathway make them more prone to cell death. Thus, this may explain why the RbTKO tumors had a much higher rate of focal CNVs than the other strains. They were able to tolerate dsDNA breaks and repair many of them but some lesions led to chromosomal lesions. This may also be true for the human retinoblastomas that express high levels of *MDMX* to suppress the p53 pathway.

### Implications for Preclinical Testing and Translational Research

Understanding the intra- and inter-specie differences is paramount when choosing the right model for the study of retinoblastoma. Unlike orthotopic xenograft models, genetic mouse models present the advantage of contributing to our understanding of the developmental processes that support tumor formation arising from a single cell. On the other hand, orthotopic xenografts from patients may more faithfully recapitulate the mechanisms by which gene deregulation occurs in the human disease. This makes orthotopic xenografts an important tool for target identification and target validation during pre-clinical trials.

The one common theme across species is inactivation of the Rb and p53 pathways. In the RbTKO tumors, they sustain *Mdm4* gain and increased Mdm4 protein expression. Therefore, targeting Mdm4 in human and mouse retinoblastoma may be relevant with respect to preclinical testing. However, there are differences in the SYK/MCL1 pathway. The upregulation of *SYK* found in virtually all human retinoblastomas was not evident in mouse retinoblastomas nor was upregulation of MCL1. Thus, preclinical testing using *SYK* or MCL1 antagonists may not provide the most relevant results for translation into clinical trials using GEMMs. Both orthotopic xenografts and GEMMs of human cancer are important for translational research but each model must be thoroughly analyzed to understand the relevant pathways that contribute to tumorigenesis and the relative role of genomic and epigenomic changes in that process to accurately interpret preclinical testing data.

## EXPERIMENTAL PROCEDURES

### Xenografts, Mouse Models of Retinoblastoma, and Cell Lines

The orthotopic xenograft (SJRB001X) has been described previously and the whole genome has been sequenced [6, 9]. Two additional orthotopic xenografts were also used in this study (SJRB002X and SJRB004X), and the primary tumors that gave rise to these xenografts have also been described previously [9]. Severe combined immunodeficiency mice (SCID) were obtained from Jackson laboratories (B6.CB17-Prkdc<scid>SzJ).

The *p107*- and *p130*-knockout mice were obtained from Dr. Tyler Jacks (Massachusetts Institute of Technology); *Chx10-Cre* mice were obtained from Dr. Connie Cepko (Harvard Medical School); *p53*^*Lox/Lox*^ and *Rb*^*Lox/Lox*^ mice were obtained from the Mouse Models of Human Cancer Consortium at the National Cancer Institute; *MDMX*^*Tg*^ mice were obtained from Dr. Guillermina Lozano (MD Anderson Cancer Center); and *p130*^*Lox/Lox*^ mice were obtained from Dr. Julien Sage (Stanford Medical School). Mice were monitored weekly for signs of retinoblastoma and anterior chamber invasion. Moribund status was defined as the point when tumor cells invaded the anterior chamber and intraocular pressure increased to the point of imminent ocular rupture. The St. Jude Laboratory Animal Care and Use Committee approved all animal procedures.

Retinoblastoma cell lines Y79, Weri1, and RB355 were cultured in RPMI medium (Lonza RPMI-1640) supplemented with 10% fetal bovine serum (Equitech Bio.), penicillin, streptomycin, and glutamate (Gibco). Cells were passaged every 3 to 4 days or when they reached 70% to 80% confluence. At the time of passage, cells were split to 20% confluence. BJ cells were grown in EMEM supplemented with 10% FBS; SJmRBL8 cells were grown in RPMI supplemented with 10% FBS; and Brca2/p53-deficient cells were grown in DMEM supplemented with 10% FBS.

### Sister Chromatid Analysis and Cytogenetics

Tumors were mechanically dissociated into a single-cell suspension in RPMI cell culture media with 10% FBS. Retinoblastoma cell lines were cultured to 60%–70% confluency. Cells were treated with colcemid (18ng/mL) for 4 hrs or 20 hrs at 37 °C. Cells were centrifuged at 900 RPM for 5 minutes. The cell pellet was resuspended in 0.075M KCl for 8 minutes at room temperature and then centrifuged at 600 RPM for 5 minutes, resuspended in 3:1 fixative (3 parts methanol: 1 part acetic acid), and incubated for 15 minutes at room temperature. The cells were added to one end of a glass slide (in upright position) in a dro*P-*wise manner by using a Pasteur pipette, allowing the cells to migrate down the slide. The slides were dried at room temperature and then stained with DAPI (80 μg/mL). Sister chromatid and spectral karyotyping (SKY) of chromosomes analyses were performed as described in Supplemental Experimental Procedures.

### Immunostaining

Cells were plated on poly-L-lysine– (Sigma) coated slides for 30 minutes and then fixed in 4% paraformaldehyde (Sigma). An anti-TP53BP1 antibody (Bethyl Labs; 1:10,000) was used followed by a biotinylated anti-rabbit secondary (Vector Labs; 1:500), VectaStain ABC (Vector Labs) and Cyanine 3 tyramide reagent (PerkinElmer) with DAPI counterstaining.

### Human Retinoblastoma Tissue Microarray Analysis

We generated a human retinoblastoma tissue microarray with 90 different tumor samples and 2 cores per tumor. The immunohistochemical staining was performed per the standard protocol on 4-μm sections of formalin-fixed, paraffinized tissue microarrays with a commercially available 53BP1 antibody (Cell Signaling; 1:100) on a Ventana automated immunostainer by using heat-induced epitope retrieval with EDTA buffer at pH 8.0 and the UltraView detection system. A “normal pattern” of expression was characterized by a homogenous nuclear staining. Complete loss or significant reduction in the intensity of nuclear staining was marked as “reduced” or “lost”, respectively. A clumped, dot-like nuclear staining was regarded as an “abnormal pattern” of expression, reflecting the activation of the DNA damage response to DNA double-strand breaks. When the abnormal pattern was observed in most of the tumor cells, it was marked as “extensive”; when it was present in only some of the tumor cells, it was marked as “focal”.

### Mouse Retinoblastoma Tissue Analysis

The immunohistochemical staining for the 3 retinoblastoma mouse models was performed on 5 different tumor samples per condition on 4-μm sections of formalin-fixed, paraffinized tissue. The commercially available TP53BP1 antibody (Bethyl Labs; 1:800) was used on a Bond Max (Leica) automated immunostainer by using heat-induced epitope retrieval with ER1 buffer (Leica) and the Bond Polymer detection system (Leica).

### Comet Assay

Alkaline single cell gel electrophoresis was performed based on the method of Singh et al[39]. Briefly, 100 μL of cells (100,000 cells/ml) suspended in PBS were mixed with 100 μL of 0.5% low melting point agarose (Sigma) and layered on CometSlides (Trevigen). The mixture was allowed to solidify at 4°C for 15 min on a metal plate. Cells were then incubated overnight at 4°C in fresh lysis buffer (2.5 M NaCl, 100 mM EDTA, 1% Triton, and 10 mM Tris, adjusted to pH 10 with NaOH). Following cell lysis, the slides were incubated at room temperature with fresh alkali buffer (300 mM NaOH, 1 mM EDTA, pH >13) for 40 min to allow DNA denaturation and unwinding. Then, the slides were run in chilled alkali buffer (300 mM NaOH, 1 mM EDTA, pH >13) at a constant electric current of 300 mA for 23 min. After electrophoresis, the slides were neutralized with three 5 min washes in 0.4 mol/L Tris-HCl (pH 7.4). Finally, the slides were fixed in 100% ethanol for 5 min and stored in the dark at room temperature. Immediately prior to imaging, comet slides were hydrated and stained by exposure to 1 mg/mL ethidium bromide for 15 min. Comets were analyzed using fluorescence based digital imaging system. Tail moments were calculated using Comet Assay Software Project (Casp) imaging software.

### DNA Methylation Analysis

DNA methylation analysis was performed using NimbleGen DNA methylation microarray services and the samples were prepared following NimbleGen's recommendations. Genomic DNA was extracted using the DNeasy Kit (Qiagen) following the manufacturing instructions including the RNeaseA treatment. 6μg of high-quality genomic DNA was digested with 24U of *MseI* (New England BioLabs) overnight at 37°C supplemented with 100ng/μl BSA and the reaction was stopped by heating the samples for 20 min at 65°C. The digested DNA was purified using QIAquick PCR Purification Kit (Qiagen) and optimal fragment size of 200-1000 bp was verified on a 2% agarose gel. 1.25μg of digested DNA was brought to a final volume of 300 μl in TE buffer (10mM Tris HCl, pH7.5; 1 mM EDTA), heat-denatured for 10 min at 95°C and immediately cooled on ice for 5 min. As control (input) DNA, 60μl of this sample were removed and stored at -20°C. To the remaining 240 μl of DNA, 60μl of 5X IP buffer (50mM Na-Phosphate, pH 7.0; 0.7 M NaCl; 0.25% Triton X-100) and 1 μg of monoclonal mouse anti 5-methyl cytidine (Abcam) was added and incubated at 4°C overnight. After overnight incubation, 48μl of a 50% slurry of Protein A agarose beads (24μl pre-washed beads resuspended in 24l of 1X IP buffer; Invitrogen) was added and incubated for 2 hr at 4°C. After washing 3 times with 1X IP buffer, the beads were digested overnight at 55°C in 250μl of digestion buffer (50 mM Tris HCl pH 8.0, 10 mM EDTA, 0.5% SDS) and 7μl Proteinase K mix (10 mg/ml). Input and IP DNA were finally phenol-chloroform purified.

### Chromatin Preparation and ChIP-on-chip

ChIP was performed by using the MAGnify Chromatin Immunoprecipitation System (Invitrogen) with some modifications. Retinae chromatin was prepared by incubating the retinae in 1% formaldehyde for 10 min at room temperature before washing them in PBS. Cross-linking was stopped by adding 1X glycine for 5 min. Retinae were then washed with PBS, dissolved in lysis buffer containing protease inhibitors (20,000 cells/μL), and incubated on ice for 30 min. Extracts were sonicated by using the Bioruptor (Diagenode) at high power until DNA fragments of 300–500 bp were formed. Sonicated chromatin was diluted 1:10 in dilution buffer. The immuno-complexes were precipitated with antibodies against polII (sc-899, Santa Cruz Biotechnologies), H3K9/14ac (49-1010, Invitrogen), H3K4me3 (49-1005, Invitrogen), H3K9me3 (49-1008, Invitrogen), or normal serum IgG (ab46540, Abcam). Precipitated complexes were reverse cross-linked, and proteins were digested with proteinase K (Invitrogen) overnight at 65 °C. Purified DNA was used for PCR and ChI*P-*on-chip analysis. ChIP experiments were run in triplicate. To obtain sufficient DNA for ChIP*-*on- chip hybridization, purified ChIP DNA was amplified by using the GenomePlex WGA kit (Sigma-Aldrich). Amplified DNA was analyzed by using 385K RefSeq Promoters Array (Roche Nimblegen).

### Gene Expression Arrays

Gene expression arrays were analyzed as described previously [9].

### FACS Analysis of DNA Content

Tumors were dissected in RPMI cell culture media containing 10% FBS and mechanically dissociated into a single cell suspension using a 1000μl pipette. Retinoblastoma cell lines were harvested at 70% confluency. 500,000 cells from the tumor cell suspension and cell lines were centrifuged at 1,500 RPM for 7 minutes at room temperature. Cell pellets were washed with PBS and centrifuged again. Cell pellets were resuspended in 0.5ml of propidium iodide solution (0.05 mg/ml propidium iodide, 0.1% (w/v) sodium citrate, 0.1% (v/v) Triton X-100).To remove RNA, 10μl of ribonuclease A (0.2 mg/ml (Calbiochem 556746) in Tris-HCl pH7.5/15mM NaCl; DNAse was heat inactivated for 15 minutes) was added to the samples for 30 minutes at room temperature and then transferred to ice. Samples were filtered through 40μm nylon mesh (Small Parts, Inc.) prior to flow cytometry (BD Biosciences Laser II).

### AGDEX analysis

Agreement of differential expression within and cross-species retinoblastoma genomics was conducted as described previously [15, 40]. The Affymetrix 430v2 array was used to profile the expression of 45101 probe-sets for 27 RBTKO, 27 MDMX, 26 p53TKO retinoblastoma samples, and 6 wt (p5) control samples. Additionally, the Affymetrix U133+2 array was used to profile the expression of 54 675 probe-sets for 57 human retinoblastoma samples and 8 control samples (fetal retina, FW18). The expression data were normalized with the MAS 5.0 algorithm.

We used the Affymetrix best-match dataset (available from www.affymetrix.com) to define 79361 pairs of ortholog-matched probe-sets across the two arrays. The best-match dataset was used to define the gene-sets for the mouse array probesets. The 1454 biological process gene-set definitions from the geneset enrichment analysis website (www.broadinstitute.org/gsea) were used for the U133+2 array.

Adaptive permutation with *B*min=100 and *B*max=10000 was used to compute *P-*values for gene-set statistics. *P-*values for individual probe-set statistics and the genome-wide dop and cosine statistics were determined using 10000 permutations.

Kruskal–Wallis test was used to compare three mouse strains (RBTKO, p53TKO and MDMX) and Wilcoxon Rank-sum test to compare two groups of RBTKO versus p53TKO, RBTKO versus MDMX, and MDMX versus p53TKO.

## SUPPLEMENTAL INFORMATION


