# Supplementary material for: Cross-species genomic and epigenomic landscape of retinoblastoma
Source: Oncotarget. 2013 May 11;4(6):844–59. doi: 10.18632/oncotarget.1051 (PMC3757242; doi:10.18632/oncotarget.1051)
Supplement: Supplementary file 1 [file oncotarget-04-844-s001.pdf]

Inventory of Supplemental Information

Number	Page
Supplemental Experimental Procedures	
Clonal Analysis of Human and Mouse Retinoblastoma	3
Sister Chromatid Analysis and Cytogenetics	3
Statistical Analysis	4
ROS Quantitation	5
aCGH	5
Exome Sequencing	6
Validation of exome sequence data	6
AGDEX analysis	7
Integrated Analysis	8
Supplementary Figures	
Supplemental Fig. 1. Genome-wide AGDEX Analysis for Mouse Retinoblastoma	10-11
Supplemental Fig. 2. Spectral Karyotype Analysis (SKY) of Mouse and Human Retinoblastomas	12-13
Supplemental Fig. 3. Clonal Analysis of Human and Mouse Retinoblastoma Cell Lines	14-15
Supplemental Fig. 4. Analysis of Oxidative Stress in Retinoblastoma	16-17
Supplemental Fig. 5. Array-Comparative Genomic Hybridization (aCGH) Analysis in Mouse Retinoblastoma	18-19

Supplementary Tables

Supplemental Table 1. Genome-wide AGDEX analysis of human retinoblastoma and mouse data 20

Supplemental Table 2. Retention Probability Estimates and Mean Absolute Ploidy Change for Tumor Samples 21

Supplemental Table 3. Micronuclei, Nuclear Blebbing and Nuclear Bridges in Retinoblastoma 22

Supplemental Table 4. Immunostaining of Primary Retinoblastoma TMA for TP53BP1 23-24

Supplemental Table 5. aCGH Whole Chromosome Events 25

Supplemental Table 6. aCGH Regional Chromosome Lesions 26

Supplemental Table 7. aCGH Recurrent Focal Lesions 27-32

Supplemental Table 8. Exome Sequencing Single Nucleotide Variations 33

Supplemental Table 9. Exome Sequencing Insertions and Deletions 34

Supplemental Table 10. Mouse Retinoblastoma Integrative Data Analysis 35-39

Supplemental Table 11. Exome Sequencing Validation Primer Sequences 40

Supplemental Table 12. Histone Mark Scoring System 41

SUPPLEMENTAL EXPERIMENTAL PROCEDURES

Clonal Analysis of Human and Mouse Retinoblastoma

Human (Y79) and mouse (SJmRBL8) retinoblastoma cell lines were seeded in 96 well plates using single-cell deposition by a high-speed cell sorter (Aria, BD Biosciences). Single cells were allowed to grow and form large clonal populations. Clones were karyotyped using the cytogenetic method described below.

Sister Chromatid Analysis and Cytogenetics

Chromosome spreads were imaged by using a Zeiss Axio Imager Z1. Images were imported into Slidebook 5 software (Intelligent Imaging Innovations, 3i, Denver, CO). The intensity profile of a line crossing the middle points of the chromatids' long arms was generated. The line profiles were then loaded into Igor Pro software (Wavemetrics Inc., Portland, OR) for fitting with a 2-peak Gaussian equation:

$$y = y_0 + A_1 \exp\left[-\left(\frac{x - x_1}{\sigma_1}\right)^2\right] + A_2 \exp\left[-\left(\frac{x - x_2}{\sigma_2}\right)^2\right]$$

A – amplitudes, σ - widths of the peaks.

The difference between centers of the peaks (i.e., x_1 and x_2) extracted from the fits was calculated as the distance between chromatids. Such measurements were made for 5 selected chromosomes per cell. Between 6 and 10 cells per sample were analyzed for each 4- or 20-hr colcemid treatment.

Spectral karyotyping (SKY) of chromosomes was performed by using a SkyPaint probe (Applied Spectral Imaging, Vista, CA) per the manufacturer's recommendations.

Probes were detected by using Applied Spectral Imaging's concentrated antibodies detection kit (CAD) as described by the manufacturer. Images were acquired with a Nikon fluorescence microscope equipped with an interferometer (Spectra Cube; Applied Spectral Imaging) and a custom-designed filter cube (Chroma Technology Corporation, Rockingham, VT). SKY analysis was performed with SKY View version 2.1 software (Applied Spectral Imaging).

For clonal studies, the chromosome spread slides were allowed to air dry for optimal banding of the chromosome with trypsin and Wright's stain. The mitotic index was adequate for a comparative chromosome analysis for all cell lines.

Statistical Analysis

Cytogenetics - Fisher's exact test was used to compare the proportion of cells with a normal genome, a doubled genome, and/or a genome with a few whole chromosomal gains or losses across cell lines. The genomic stability of cell line clones was measured by estimating the probability that the clone retained the number of chromosomes present in its parent cell. This retention estimate was computed by first determining the proportion of clone cells with the same number of chromosomes as each parental cell and then averaging these proportions over all the parental cells. The Wilcoxon rank sum test was used to compare the retention probability estimates across two groups.

Sister chromatid cohesion - The inter-chromatid distances varied substantially between individual cells within the same cell population. Therefore, to perform pairwise comparisons across cell populations, the mean inter-chromatid distance was computed for each cell and these within-cell means and the Wilcoxon rank-sum test was used to

perform pairwise comparisons of the cell-mean values across cell populations as previously described (Pounds and Dyer, 2008).

ROS Quantitation

Intracellular reactive oxygen species (ROS) were analyzed by flow cytometry using dichlorofluorescein diacetate (DCFH-DA; Sigma) as a specific dye probe which fluoresces upon oxidation by ROS. Cells were seeded at $1 \cdot 10^5$ cells per 35 mm dish. Cells loaded with DCFH-DA (50 μg/ml) with light exclusion for 60 min were washed three times with PBS. Intracellular accumulation of fluorescent DCF-DA was measured (10,000 cells each) by flow cytometry.

aCGH

A genome-wide microarray (G4838A, Agilent Mouse CGH 1x1M), consisting of 60mer *in situ* synthesized oligonucleotides, was designed and manufactured by Agilent Technologies (Santa Clara, CA). This array contained 963,261 unique biological probes spaced across the mouse genome. Array hybridization was performed according to the manufacturer's recommended protocols. In brief, 1.5 μg of each genomic DNA sample was labeled using the Agilent's ULS Labeling kit. Hybridization was carried out in an Agilent oven at 65°C for 40 hours at 20 rpm, followed by standard wash procedures. The microarray was then scanned in an Agilent scanner at 3 μm resolution, and the array data was extracted using the default CGH settings with LOWESS normalization of Agilent Feature Extraction Software (v10.5.1.1). Tumor DNA was being compared to control DNA from tail of the same animal. The circular binary segmentation method implemented in Partek software (Partek Inc., St. Louis, MO) was applied for copy

number variation calling. Aberration calls were assigned to those segments with at least 5 markers (probes), p-value less than 0.05, and mean log2 ratio greater than 0.15 or less than -0.25. In addition, the calls on whole chromosome gain or loss were made for those aberrant segments having size greater than 80% of that of the chromosome, with mean log2 ratio greater than 0.1 or less than -0.12, respectively. The recurrent gains and losses at genomic location level and gene level, except for the sex chromosomes, were further summarized in three categories (whole chromosome, large chromosomal segment (>3Mb), and focal lesion (>20Kb and <3Mb)) among each of the three tumor groups.

Exome Sequencing

Whole genome amplified DNA was prepared using the repli-G WGA kit (Qiagen). Whole exon enrichment was accomplished using the SureSelect XT Mouse All Exon Kit (Agilent) as directed except 4μg of WGA material was substituted for 3μg of genomic DNA. Sequencing was performed on an Illumina HiSeq 2000 with V3 flow cells, using the paired end 100 cycle protocol, with the samples multiplexed to an equivalent of 3 per lane. The resulting data files were demultiplexed and converted to FASTQ files using CASAVA 1.8.2 (Illumina) and mapped using BWA to the mouse reference build MM9.

Validation of exome sequence data

PCR amplification using TAQGOLD 360 (ABI) of regions containing putative variants was performed with synthetic oligos (IDT) designed (PRIMER 3) to match sequence flanking the putative variants (primers and locations listed in Sup. Table 11). Both tumor and paired normal samples were amplified and pooled separately. Amplicon libraries were prepared using the Nextera XT (Illumina) sample preparation kit and sequenced

using a MiSeq following the 150 cycle paired end protocol. Variants were identified by running MiSeq reporter or Bambino software, and scored as either somatic or germline.

AGDEX analysis

Agreement of differential expression within and cross-species retinoblastoma genomics was conducted as described previously (Pounds et al., 2011a; Pounds et al., 2011b). The Affymetrix 430v2 array was used to profile the expression of 45101 probe-sets for 27 RBTKO, 27 MDMX, 26 p53TKO retinoblastoma samples, and 6 wt (p5) control samples. Additionally, the Affymetrix U133+2 array was used to profile the expression of 54 675 probe-sets for 57 human retinoblastoma samples and 8 control samples (fetal retina, FW18). The expression data were normalized with the MAS 5.0 algorithm.

We used the Affymetrix best-match dataset (available from www.affymetrix.com) to define 79361 pairs of ortholog-matched probe-sets across the two arrays. The best-match dataset was used to define the gene-sets for the mouse array probesets. The 1454 biological process gene-set definitions from the geneset enrichment analysis website (www.broadinstitute.org/gsea) were used for the U133+2 array.

Adaptive permutation with Bmin=100 and Bmax=10000 was used to compute P-values for gene-set statistics. P-values for individual probe-set statistics and the genome-wide dop and cosine statistics were determined using 10000 permutations.

Kruskal–Wallis test was used to compare three mouse strains (RBTKO, p53TKO and MDMX) and Wilcoxon Rank-sum test to compare two groups of RBTKO versus p53TKO, RBTKO versus MDMX, and MDMX versus p53TKO.

Integrated Analysis

First, mRNA expression from 430v2 Affymetrix arrays was RMA summarized in Partek Genomics Suite 6.6 (St. Louis Mo) and compared between the RBTKO tumors and P5 wt retinae controls employing the unequal variance t test and log2ratio. Next, peak scores for methylation data provided by Nimblegen arrays were compared between RBTKO and P5 wt samples by unequal variance t test and mean peak difference for each observation. Finally, Nimblegen ChIP-chip data was collated by histone mark and evaluated for RBTKO and control samples and scores by the following protocol. For any histone class for each genotype and gene, a signal must be present in at least 2 of the 3 samples for it to be considered present. If histone marks are present only in RBTKO samples then the mark is given a 1. If they are absent in both or present in both tumor and control, then a 0 is given. When only the control peak is present, it is assigned a -1. The calls for each histone class are then combined and scored jointly (Sup. Table 12).

The three data types were joined by refseq id or by genesymbol. Data that did not match to expression data was excluded. Data was filtered by removing all probesets that did not have at least one expression value greater than 5 or |fold change RBTKO vs wt | > 2. Repeated measures within a gene were removed by selecting the probeset with the greatest |logratio|. Repeated measures in ChIP-chip were removed by retaining the greatest |combined score – 4|. For repeated measures of methylation data the greatest mean diff was retained.

Prior to ranking p values for methylation, data were inferred to be 1 with mean diff of zero for any genes symbols where that data was not observed. Likewise, for any gene without ChIP-chip data a combined score of 4 was imposed. In order to achieve a two metric for p-values from expression and methylation scores were produced. The

scores were produced by taking the –log10(pvalue) and then multiplying data with logratios below zero by -1. This creates a dispersed two tail p value derived metric. Care was then taken to coherently rank the scores such that expression was ranked high to low, methylation was ranked hypo to hyper methylated and histones were ranked unmarked to highly marked in RBTKO. The pvalue metrics were multiplied by -1 if not positively correlated to their magnitude change. The ranks between expression and the methylation marks were negatively correlated. In this way, each magnitude difference value was two sided with genes of interest at the extremes of the distribution. This process yielded 5 ranks, which were then additively combined by the following formula for each gene:

Combined rank = (logratio WT/TKO expression rank + pvalue expression metric rank)/2 + combined histone score rank + (rank mean diff TKO- WT + pvalue methylation metric rank)/2.

The 5 rank lists were each randomly sampled with replacement to create a new combine rank 1M times. For each gene the observed test statistic was then compared to the population of bootstrapped test values to determine the p value. The Benjamini and Hochberg(Benjamini and Hochberg, 1995) method for controlling false discovery rate was implemented in STATA/MP 11.2. This entire process was then applied to the previously published retinoblastoma human data (Zhang et al., 2012), so that the results could be compared (Sup. Fig. 5).

Supplemental Figure 1. Genome-wide AGDEX Analysis for Mouse Retinoblastoma.

Agreement of differential expression (AGDEX) for the mouse comparison (x-axis) and human comparison (y-axis) for each of the 79361 probe-set pairs for (A) RbTKO, (B) MDMX, and (C) p53TKO mouse retinoblastoma models.

A

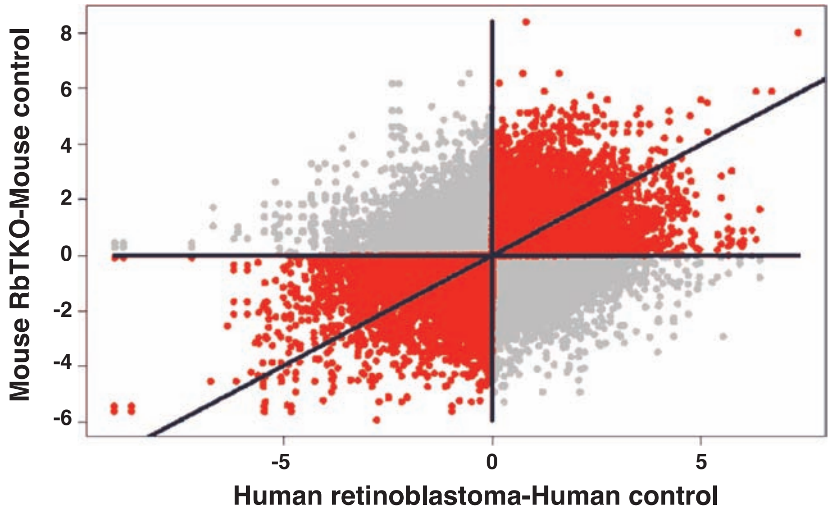

cos=0.23, cos.pva A=8e-04, cos.pva B=7e-04, dop=0.14, dop.pva A=2e-04, dop.pva B=0.0021

B

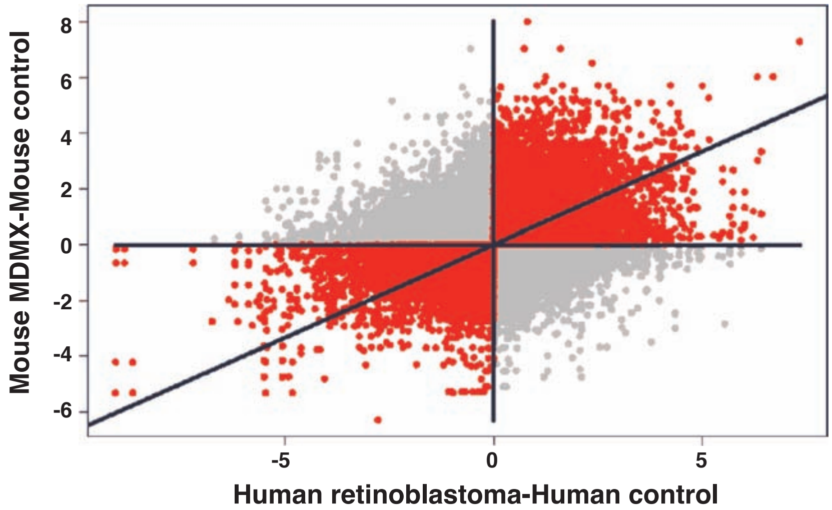

cos=0.24, cos.pva A=0.0342, cos.pva B=0.0019, dop=0.14, dop.pva A=0.0026, dop.pva B=0.0026

C

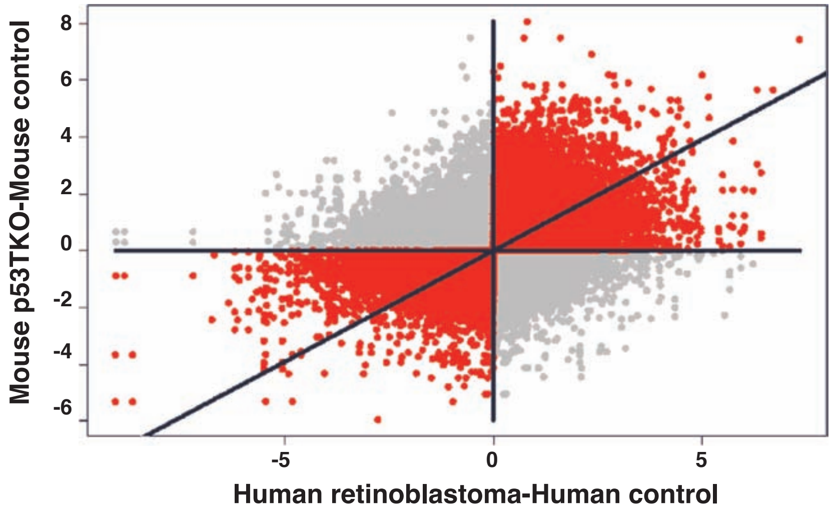

cos=0.23, cos.pva A=0.0564, cos.pva B=0.004, dop=0.13, dop.pva A=0.0107, dop.pva B=0.0041

Supplemental Figure 2. Spectral Karyotype Analysis (SKY) of Mouse and Human Retinoblastomas

Representative SKY image of (A) p53TKO (*Chx10-Cre;Rb*$^{Lox/Lox}$*;p107*$^{-/-}$*;p53*$^{Lox/Lox}$), (B) MDMX (*Chx10-Cre;Rb*$^{Lox/Lox}$*;p107*$^{-/-}$*;MDMX*^{Tg}), (C) RBTKO (*Chx10-Cre;Rb*$^{Lox/Lox}$*;p107*$^{-/-}$*;p130*$^{Lox/Lox}$) mouse retinoblastomas and (D-E) human retinoblastoma xenografts.

p53TKO

A

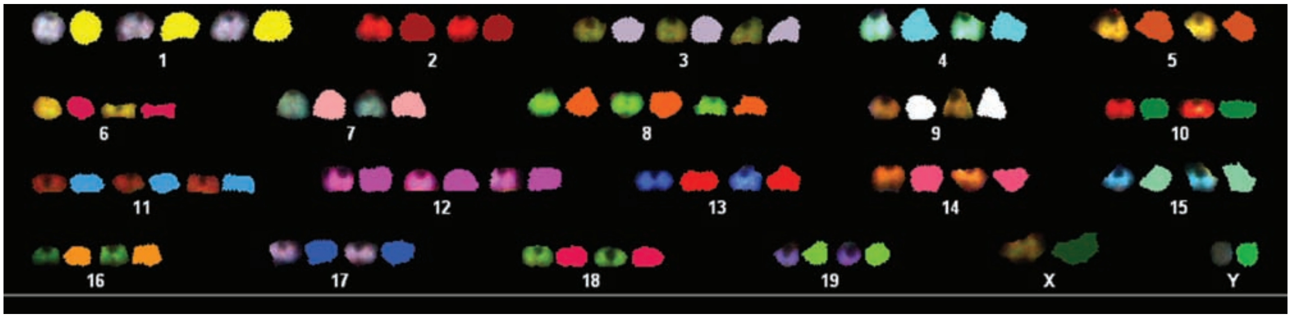

MDMX

B

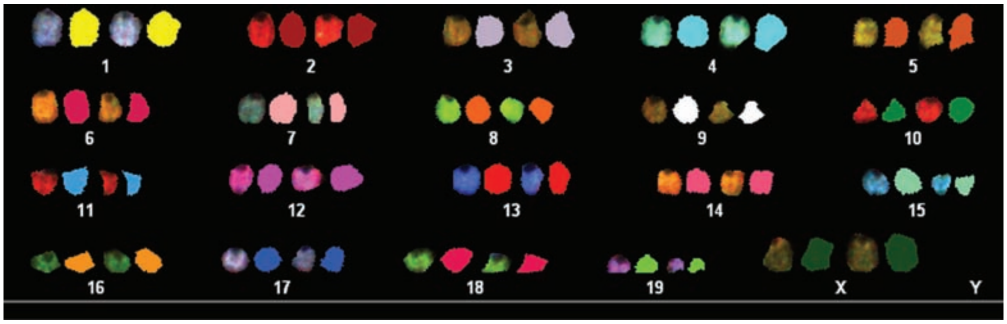

RbTKO

C

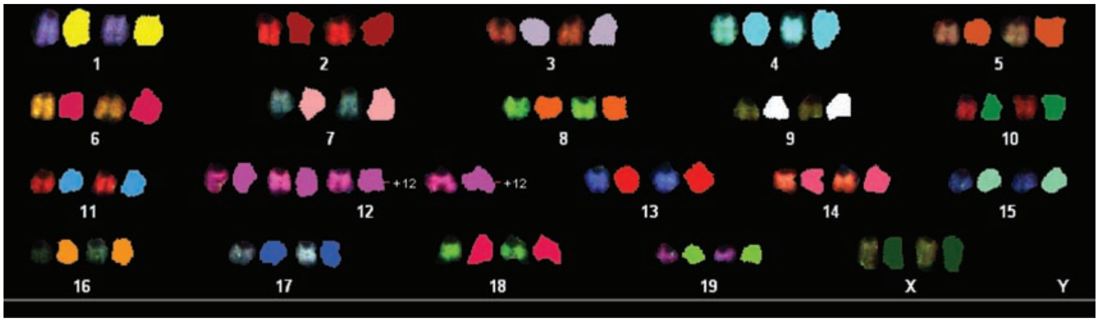

SJRB002X

D

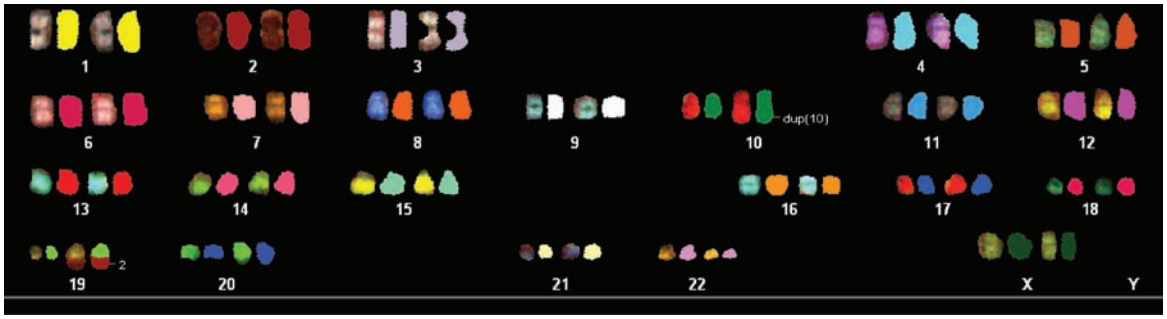

SJRB004X

E

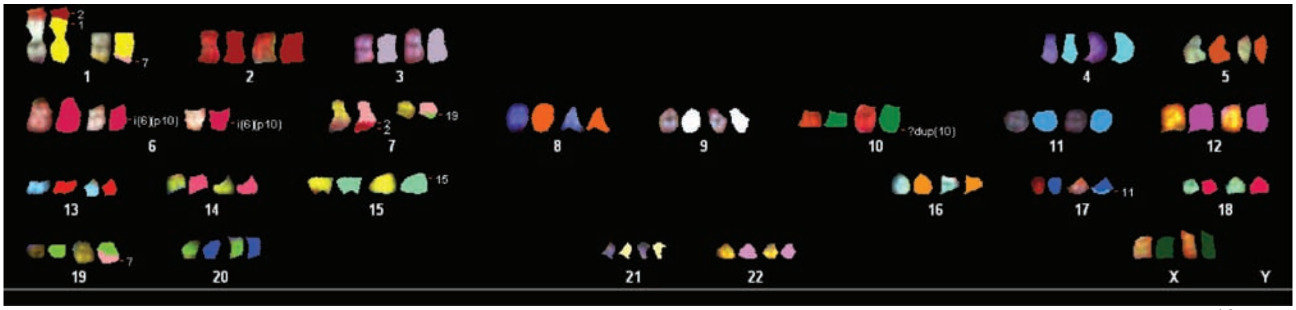

Supplemental Figure 3. Clonal Analysis of Human and Mouse Retinoblastoma Cell Lines

(A, B) Representative Wright's stained chromosomal spread for the Y79 parental cell line used for the clonal analysis and one of the 9 clones analyzed in this study that were derived from a single cell. (C, D) Representative Wright's stained chromosomal spread for the SJmRBL8 mouse retinoblastoma cell line for the parental cell and one of the 9 clones derived from a single cell showing multiple chromosomal gains/losses (arrows). (E) Histogram of the average chromosomal number variation from the parental cell for 3 independent clones for the human retinoblastoma cell line (Y79, gray) and the mouse retinoblastoma cell line (SJmRBL8, black.)

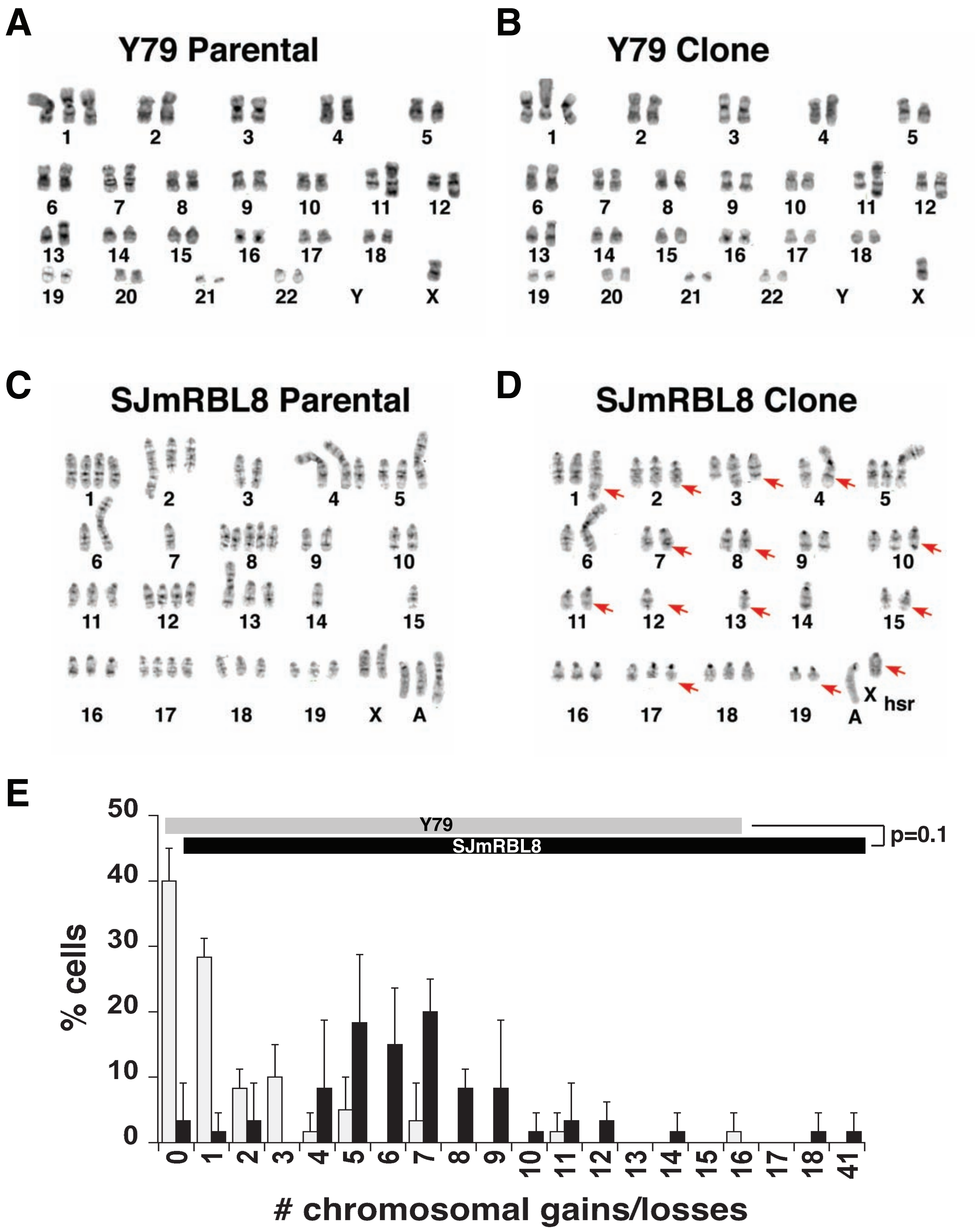
A
Y79 Parental
1 2 3 4 5
6 7 8 9 10 11 12
13 14 15 16 17 18
19 20 21 22 Y X
B
Y79 Clone
1 2 3 4 5
6 7 8 9 10 11 12
13 14 15 16 17 18
19 20 21 22 Y X
C
SJmRBL8 Parental
1 2 3 4 5
6 7 8 9 10
11 12 13 14 15
16 17 18 19 X A
D
SJmRBL8 Clone
1 2 3 4 5
6 7 8 9 10
11 12 13 14 15
16 17 18 19 A X hsr
E
Y79
SJmRBL8
p=0.1
% cells
0 10 20 30 40 50
0 1 2 3 4 5 6 7 8 9 10 11 12 13 14 15 16 17 18 41
chromosomal gains/losses

Supplemental Figure 4. Analysis of Oxidative Stress in Retinoblastoma

(A) Histogram of relative increase in DCF-DA fluorescence in BJ cells following treatment with different concentration of hydrogen peroxide for 24 hours with or without catalase. Data are plotted relative to untreated samples. (B) Proportion of Annexin V immunopositive BJ cells as measured by flow cytometry for a subset of the samples in (A). (C) Representative plots of the DCF-DA flow cytometry and the Annexin V flow cytometry. (D-F) Similar data for the SJRB001X xenograft analyzed side-byside with BJ cells. (G-J) Histogram of relative increase in DCF-DA fluorescence in mouse normal cells (MEF) and primary mouse retinoblastomas (RBTKO, MDMX, p53TKO).

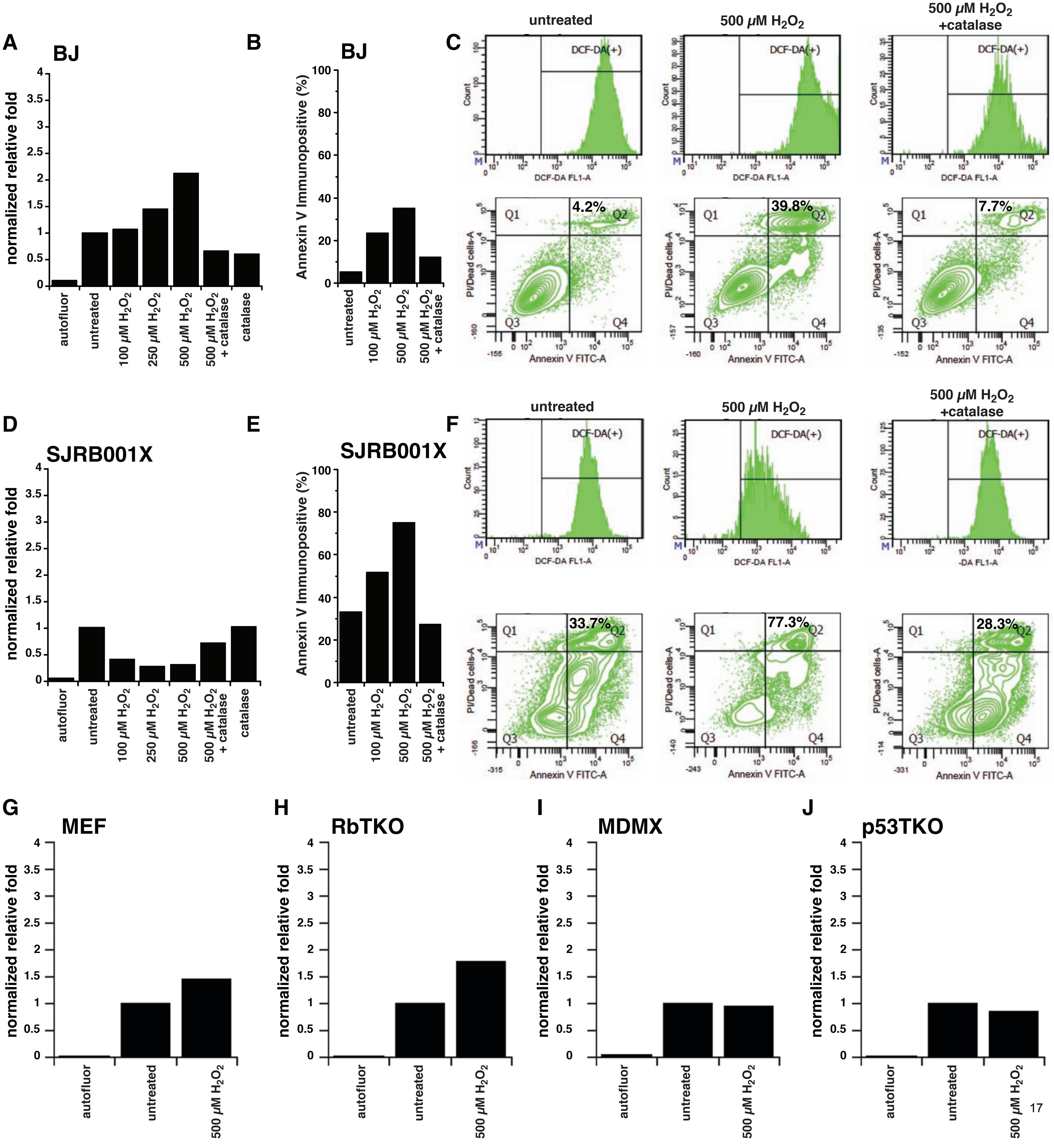
A
BJ
normalized relative fold
autofluor
untreated
100 μM H2O2
250 μM H2O2
500 μM H2O2
500 μM H2O2 + catalase
catalase
B
BJ
Annexin V Immunopositive (%)
C
untreated
500 μM H2O2
500 μM H2O2 +catalase
DCF-DA(+)
DCF-DA FL1-A
Count
4.2%
39.8%
7.7%
Q1
Q2
Q3
Q4
PI/Dead cells-A
Annexin V FITC-A
D
SJRB001X
E
SJRB001X
F
33.7%
77.3%
28.3%
G
MEF
H
RbTKO
I
MDMX
J
p53TKO

Supplemental Figure 5. Array-Comparative Genomic Hybridization (aCGH) Analysis in Mouse Retinoblastoma

aCGH analysis of six RbTKO (TKO), six MDMX (MDMX), and six p53TKO (P53) independent retinoblastoma tumor samples. Red lines denote areas of the genome that are amplified and blue lines denote deletions.

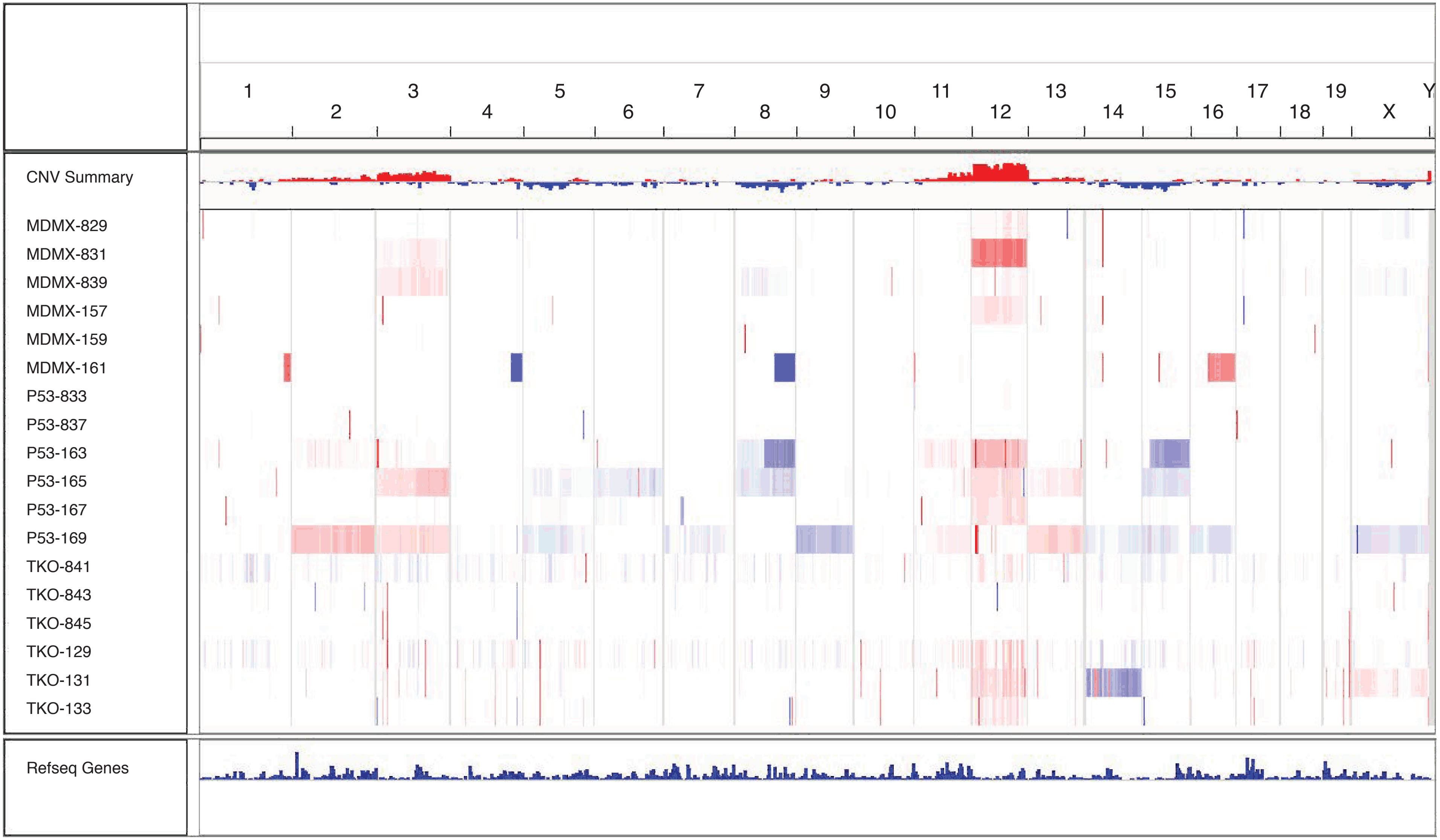
1
2
3
4
5
6
7
8
9
10
11
12
13
14
15
16
17
18
19
X
Y
CNV Summary
MDMX-829
MDMX-831
MDMX-839
MDMX-157
MDMX-159
MDMX-161
P53-833
P53-837
P53-163
P53-165
P53-167
P53-169
TKO-841
TKO-843
TKO-845
TKO-129
TKO-131
TKO-133
Refseq Genes

Supplemental Table 1. Genome-wide AGDEX analysis of human retinoblastoma and mouse data

	RBTKO			p53TKO			MDMX		
**stat.name**	**stat.value**	**human.pval**	**mouse.pval**	**stat.value**	**human.pval**	**mouse.pval**	**stat.value**	**human.pval**	**mouse.pval**
**cos**	0.232455	8.00E-04	7.00E-04	0.233693	0.0564	0.004	0.236306	0.0342	0.0019
**dop**	0.139419	2.00E-04	0.0021	0.132405	0.0107	0.0041	0.139381	0.0026	0.0026

Supplemental Table 2: Retention Probability Estimates and Mean Absolute Ploidy Change for Tumor Samples

Species	Sample ID	[1]Retention	[2]MAPC	[3]MAPC p (SJRB001X)	[4]MAPC p (RbTKO)	[5]MAPC p (MDMX)
human	SJRB001X	0.7500	0.4167		0.0711	<0.0001
mouse	p53TKO	0.3478	15.8261	0.0009	0.0067	0.9924
mouse	mdmx	0.0000	3.5385	<0.0001	<0.0001	
mouse	RbTKO	0.4773	0.7045	0.0711		<0.0001

[1]Retention: retention probability estimate
[2]MAPC: mean absolute ploidy change
[3]MAPC probability relative to SJRB001X
[4]MAPC probability relative to RbTKO
[5]MAPC probability relative to MDMX

Supplemental Table 3. Micronuclei, nuclear blebbing and nuclear bridges in retinoblastoma.

Sample	Micronuclei Counts	(%)	Nuclear Bridges Counts	(%)	Nuclear Blebbing Counts	(%)
SJRB001X	2/264	(0.8%)	0/264	(0.0%)	2/264	(0.8%)
SJRB002X	0/272	(0.0%)	0/272	(0.0%)	0/272	(0.0%)
Y79	1/293	(0.3%)	0/293	(0.0%)	4/293	(1.4%)
Weri1	0/253	(0.0%)	0/253	(0.0%)	1/253	(0.4%)
RB355	1/253	(0.4%)	0/253	(0.0%)	1/253	(0.4%)
BJ	0/250	(0.0%)	0.250	(0.0%)	0.250	(0.0%)
RbTKO	1/252	(0.4%)	1/252	(0.4%)	5/252	(2.0%)
MDMX	2/251	(0.8%)	0/251	(0.0%)	3/251	(1.2%)
P53TKO	1/253	(0.4%)	2/253	(0.8%)	4/253	(1.6%)
SJmRBL8	4/252	(1.6%)	0/252	(0.0%)	1/252	(0.4%)
MEF	1/262	(0.4%)	1/262	(0.4%)	9/262	(3.4%)

Supplemental Table 4. Immunostaining of Primary Retinoblastoma TMA for TP53BP1.

Sample	53BP1 IHC pattern of expression	Sex	Ethnicity	Age (m)	[1]Stage	[2]Differentiation	[3]Invasion	[4]ON	Seeds	RB1 germline
S-00-1159-A3	dot-like nuclear expression (focal)									
S-00-158M	normal pattern	F	Black	28	E, Vb	undiff, nec	SR	N	Y	negative
S-00-184-M	normal pattern	M	White	16	E, Vb	diff, HW, FW	Ch, SR	ant	Y	No; polymorphisms
S-01-564-M	normal pattern	M	Black	19	E, Vb	undiff, nec, FW	Ch++	post	Y	POSITIVE
S-01-630-M	normal pattern	M	White	52	D, Vb	HW, FW	N	ant	Y	No; polymorphisms
S-02-1123-1S	normal pattern	M	Black	15	E, Vb	nec, FW, HW	Ch++	post	Y	POSITIVE
S-02-1529-M	dot-like expression (rare)	F	White	18	D, Vb	diff	Ch++	n	Y	negative
S-02-218-M	normal pattern	M	White	20	E, Vb	undiff, nec, calc	SR	ant	Y	No; polymorphisms
S-03-111-1M	dot-like nuclear expression (focal)	M	Black	58	E, Vb	FW, HW, nec	Ch	at	Y	No; polymorphisms
S-03-1450-A2	normal pattern	M	Black	40	E, Vb	undiff	N	ant		n/a
S-03-1468-A2	normal pattern	F	Black	43	B, Vb	undiff	N	ant	Y	No; polymorphisms
S-03-1731-A1	reduced expression	F	White/Mexican	26	E, Vb	nec, undiff, HW	SR	n	Y	No; polymorphisms
S-03-174-1S	missing	F	White	30	E, Vb	HW, FW	Ch	n		No; polymorphisms
S-03-285-1M	dot-like expression (rare)	M	White	38	E, Vb	undiff	N	ant		n/a
S-03-470-1S	normal pattern	M	White	7	E, Vb	?	Ch++, SR	at		POSITIVE
S-04-005-A2	normal pattern	M	Black	45	E, Vb	nec, HW	CB, Ch	ant	Y	No; polymorphisms
S-04-118-A2	normal pattern/dot-like nuclear expression (extensive)	F	White	35	D, Vb	diff	Ch	ant		No; polymorphisms
S-04-1221-A2	normal pattern	F	Black	11	E, Vb	diff, glaucoma	Ch	post	Y	POSITIVE
S-04-1533-A3	dot-like nuclear expression (rare)	F	Black	11	D, Vb	?	N	n	Y	n/a
S-04-407-A2	reduced expression	M	white/Cherokee	53	?	focal nec	Ch	Post	?	n/a
S-04-426-A2	normal pattern	F	White	12	D, Vb	min nec	N	ant	Y	No; polymorphisms
S-04-449-A2	normal pattern	F	White	19	D, Vb	?	Ch	n	Y	No; polymorphisms
S-04-502-A1	normal pattern with rare dot-like nuclear expression	M	white	16	?	Diff	N	N	Y	No; polymorphisms
S-04-988-A2	dot-like nuclear expression (focal)	F	White	8	D, Vb	HW, FW	SR	n	Y	No; polymorphisms
S-05-053-A2	normal pattern	M	White	10	E, Vb	?	Ch	ant		negative
S-05-1115-A3	dot-like nuclear expression (extensive)	M	black	41	Vb	FW, HW, nec	Ch, Sc, AC, I	at	Y	No; polymorphisms
S-05-1147-A3	dot-like nuclear expression (rare)	F	white	37	Vb	undiff, fleur, nec	N	N	Y	POSITIVE
S-05-1236-A2	normal pattern	M	white/mexican	11	Va	FW, HW	Ch	post	N	POSITIVE
S-05-1269-A2	dot-like nuclear expression (focal)	F	Black	26	Vb	undiff, fleur	AC, SR	ant	Y	POSITIVE
S-05-1342-A2	normal pattern/dot-like nuclear expression (focal)	F	White	77	Vb	min nec	AC, SR	post	Y	No; polymorphisms
S-05-138-A1	dot-like nuclear expression (focal)	M	White	40	?	partial nec	Ch	post	?	No; polymorphisms
S-05-1457-A2	dot-like expression (rare)	M	Black	19	Vb	?	N	ant	Y	No; polymorphisms
S-05-512-A3	normal pattern/dot-like nuclear expression (extensive)	M	Black	10	Vb	FW, nec	N	ant	Y	negative
S-05-640-A2	necrotic tissue	F	White	19	Vb	nec	CB	N	Y	No; polymorphisms
S-05-793-A1	dot-like nuclear expression (extensive)	M	Black	16	Vb	diff, FW, nec	N	ant	Y	No; polymorphisms
S-05-993-A2	dot-like nuclear expression (rare)	F	White	34	Vb		Ch	at	y	negative
S-06-010-A2	dot-like nuclear expression (extensive)	F	black	108	Vb		N	at	y	POSITIVE
S-06-1026-A2	dot-like expression (rare)	M	Puerto Rico/white	28	Vb	diff, FW, nec	Ch, SR	ant	Y	Negative
S-06-1758-A2	necrotic tissue	F	black	2	Vb	nec, rupture	N	n	y	POSITIVE
S-06-1776-A2	dot-like nuclear expression (focal)	F	Multiple	81	Vb	undiff, nec	CB, I, AC, Scl	n	y	No; polymorphisms
S-06-1871-C3	normal pattern/dot-like nuclear expression (focal)	F	White	24	Vb	undiff	I, AC, CB, Scl	at	y	No; polymorphisms
S-06-265-A1	normal pattern	F	hispanic/white	67	Vb	diff, HW, FW	N	post	Y	POSITIVE
S-06-705-A3	dot-like expression (rare)	M	White	45	Va	un/diff, FW, HW	Ch, SR	at	n	No; polymorphisms
S-07-1340-A3	dot-like nuclear expression (focal)	F	Black	22	D, Vb	undiff, HW	SR	at	Y	No; polymorphisms
S-07-1412-A3	dot-like nuclear expression (focal)	M	black	8	D, Vb	diff, HW, FW	Ch, SR	n	y	No; polymorphisms
S-07-1483-A3	reduced with dot-like nuclear expression (focal)	F	Black	47	D, Vb	Diff, FW	Ch	N	Y	No; polymorphisms
S-07-284-A2	normal pattern	M	White	45	E, Vb	undiff	N	N	Y	No; polymorphisms
S-07-295-A3	reduced expression	F	Black	37	Vb	undiff	Ch++, I, Sc	Post	Y	No; polymorphisms
S-07-462-1A	dot-like nuclear expression (focal)	F	White	7	Va	undiff	Ch	N	Y	No; polymorphisms
S-07-503-A1	normal pattern	F	White	15	Vb	undiff, FW, HW	Ch, SR	ant	y	No; polymorphisms
S-07-934-A3	normal pattern	F	white	11	E, Vb	undiff, FW, HW	Ch, SR	ant	Y	No; polymorphisms
S-07-938-A3	dot-like nuclear expression (focal)	M	white	34	E, Vb	Undiff	Ch, SR	n	y	No; polymorphisms
S-07-967-A3	dot-like nuclear expression (extensive)	M	white	24	D, Vb	undiff, HW	Ch, SR	n	y	negative
S-08-1188-A3	dot-like nuclear expression (extensive)	M	hispanic/white	6	Va	undiff/diff, FW, ne	Ch++, SR	post	N	No; polymorphisms
S-08-1244-A2	normal pattern/dot-like nuclear expression (focal)	M	White	33	Vb	undiff, nec	N	N	Y	No; polymorphisms
S-08-1713-A3	dot-like nuclear expression (focal)	M	Black	36	Vb	diff	Ch	ant	Y	No; polymorphisms
S-08-178-A2	dot-like nuclear expression (focal)	M	white	4	D, IV b	Undiff, FW	SR	n	y	POSITIVE
S-08-1859-A2	dot-like nuclear expression (focal)	F	White	32	D, Va	undiff	SR	ant	N	No; polymorphisms
S-08-1887-A3	dot-like nuclear expression (extensive)	F	Black	13	E, Vb	undiff, FW, HW	Ch, SR	N	Y	No; polymorphisms
S-08-1924-A3	dot-like nuclear expression (focal)	M	Other (hispanic)	29	Vb	diff, HW, FW	Ch, SR	ant	Y	POSITIVE
S-08-258-A3	largely necrotic, reduced with dot-like nuclear expression (focal)	M	black	48	E, Vb	Undiff	Ch, SR	n	y	No; polymorphisms
S-08-286-A3	dot-like nuclear expression (extensive)	F	Am Indian/AK	67	D, Vb	undiff	SR	ant	y	No; polymorphisms
S-08-461-A3	dot-like nuclear expression (focal)	M	white	13	Va	undiff	Ch++	at	N	No; polymorphisms
S-08-639-A4	normal pattern	M	Black	75	Vb	diff, HW, FW	N	ant	Y	No; polymorphisms
S-08-77-A3	dot-like nuclear expression (extensive)	M	White	13	Va	HW, FW, nec	SR, Ch, Scl	at	y	POSITIVE
S-08-798-A4	dot-like nuclear expression (extensive)	M	Black	10	D, Vb	undiff, FW, HW	Ch	N	Y	No; polymorphisms
S-09-1019-A4	dot-like nuclear expression (focal)	M	White	28	E, Vb	undiff, nec	Ch++	post	Y	No; polymorphisms
S-09-1154-A3	dot-like nuclear expression (focal)	M	Black	13	Vb	undiff, FW	Ch, SR	ant	Y	No; polymorphisms
S-09-1159-A3	reduced expression	F	White	9	Vb	undiff, FW	Ch++	Post	Y	No; polymorphisms

S-09-1238-A4	dot-like nuclear expression (extensive)	F	white	22	Vb	undiff, FW	SR	at	Y	No; polymorphisms
S-09-1368-A4	dot-like nuclear expression (extensive)	M	Black	26	E, Vb	undiff/diff, FW, H	SR	ant	Y	No; polymorphisms
S-09-1436-A3	normal pattern/dot-like nuclear expression (extensive)	F	hispanic/"white"	17	E, Vb	undiff, HW, FW	N	ant	Y	No; polymorphisms
S-09-1641-A3	dot-like expression (extensive)	F	American Indian	13	E, Vb	undiff, FW, HW, n	Ch, SR	ant	Y	POSITIVE
S-09-1675-A2	dot-like nuclear expression (extensive)	M	White	43	E, Vb	undiff, nec	Ch, SR	ant	Y	No; polymorphisms
S-09-29-A3	dot-like nuclear expression (focal)	M	unknown	7	E, Va	diff, HW, FW	AC, SR	post	N	No; polymorphisms
S-09-33-A2	necrotic tissue	F	White	3.5	E, Vb	nec	Ch++	at	Y	POSITIVE
S-09-473-A3	reduced expression	F	White	23	E, Va	undiff	Ch++, Sc	at	N	n/a
S-89-706-1	normal pattern	M	White	28	D, Vb		N	ant	y	n/a
S-90-982-1	normal pattern	M	Black	20	D, Vb	undiff, nec	N	ant	y	No; polymorphisms
S-92-1534-1M	lost	F	White	14	D, Vb	undiff, FW	Ch++	n	y	POSITIVE
S-92-291-1M	normal pattern/dot-like nuclear expression (focal)	F	black	6	D, IIIb	diff, rosettes	N	n	y	n/a
S-92-556-1I	normal pattern	F	Am Ind/Spain/Mex	2	E, Va	diff, FW	SR, Ch++	n	y	POSITIVE
S-93-1638-1I	normal pattern	M	White	7	E, Va	FW	Ch, SR	ant	n	POSITIVE
S-93-745-1A1	dot-like nuclear expression (focal)	F	White	44	D, Vb	diff, fleurettes	I, Ch	ant	y	No; polymorphisms
S-93-849-1M	dot-like nuclear expression (rare)	F	White	24	D, Vb	undiff, nec	SR	post	y	n/a
S-95-181-1M	normal pattern	M	White	15	D, Va	diff, FW	SR	at	n	POSITIVE
S-96-135-1I	dot-like nuclear expression (extensive)	F	Black	13	E, Vb	diff, FW, nec	Ch, SR	ant	y	POSITIVE
S-97-423-M	normal pattern	M	White	29	D, Vb	undiff	Ch++, SR	n		n/a
S-98-1069-1M	reduced expression	M	black	31	E, Va	undiff	Ch++, SR	post	n	n/a
S-98-1217-M	normal pattern	F	Black	27	E, Va	diff, FW	Ch, SR	post	n	n/a
S-99-499-1S	normal pattern with rare dot-like nuclear expression	M	white	29	E, Vb	diff/undiff	SR	n	y	n/a
S-99-898-1M	normal pattern/dot-like nuclear expression (focal)	F	Black	23	D, Va	diff, FW	Ch, CB	ant	n	No; polymorphisms
S-99-951-M	dot-like nuclear expression (focal)	F	white	36	C, IIIb	undiff	N	ant	?	No; polymorphisms
S00-184-M	normal pattern	M	white	16	E, Vb	diff	Ch, SR	ant	y	No; polymorphisms

[1] International retinoblastoma stage is listed first and Reese-Ellsworth stage is listed second
[2] Retinoblastoma growth is characterized as exophytic, endophytic or a combination of the two
[3] Anterior chamber (AC), iris (I), ciliary body (CB), choroidal (Ch), massive choroidal (Ch++), subretinal (SR), scleral (Sc) invasion was scored for each sample
[4]Optic nerve invasion scored as none (N), anterior (ant), at, or posterior (post) to lamina cribosa, or present at cut end of the optic nerve (end)

Diff = differentiated
Fleur = fleurettes
FW = Flexner-wintersteiner rosettes
HW = Homer Wright rosettes
Nec = necrosis
Undiff = undifferentiated

Supplemental Table 5. aCGH whole chromosome events.

Model	Sample	Amplification	Deletion	Human retinoblastoma synteny
**RbTKO**	TKO-841	--	--	--
	TKO-843	--	--	--
	TKO-845	--	--	--
	TKO-129	chr12	--	14q32
	TKO-131	chr12	chr14	14q32 / 13q14
	TKO-133	chr12	--	14q32
	Recurrent	*chr12*	--	*14q32*
**MDMX**	MDMX-829	--	--	--
	MDMX-831	chr3, chr12	--	--, 14q32
	MDMX-839	chr3	--	--
	MDMX-157	chr12	--	14q32
	MDMX-159	--	--	--
	MDMX-161	--	--	--
	Recurrent	*chr3, chr12*	--	--, *14q32*
**p53TKO**	P53-833	--	--	--
	P53-837	--	--	--
	P53-163	chr2, chr11, chr12	chr8, chr15	--, 7p12, 14q32, --, --
	P53-165	chr3, chr12, chr13	chr5, chr6, chr8, chr15	--, 14q32, 6p, --, --, --, --
	P53-167	chr12	--	14q32
	P53-169	chr2, chr3, chr11, chr13	chr5, chr7, chr9, chr14, chr15, chr16	--, -- , 7p21, 6p, --, --, --, 13q14, --, --
	Recurrent	*chr2, chr3, chr11, chr12, chr13*	*chr5, chr8, chr15*	--, --, *7p21, 14q32, 6p*, --, --, --

Supplemental Table 6. aCGH regional chromosome lesions.

Model	Sample	Amplification	Deletion	Human retinoblastoma synteny
**RbTKO**	TKO-841	chr12:21386244-31120879	--	--
		chr12:53222949-57198172		--
		chr12:70246090-75030786		--
		chr12:76247215-85856306		--
		chr12:85961984-90674618		--
		chr12:98866622-102398632		--
		chr12:108896847-114742841		14q32
	TKO-843	--	--	--
	TKO-845	--	--	--
	TKO-129	chr4:128446658-134101300	--	--
		chr5:114783390-117888782		--
		chr5:120596817-124824557		--
		chr11:113586761-120496749		--
	TKO-131	--	--	--
	TKO-133	--	--	--
	Recurrent	--	--	--
**MDMX**	MDMX-829	chr12:70463725-90559950	--	--
		chr12:100446039-121257272		14q32
	MDMX-831	--	--	--
	MDMX-839	--	--	--
	MDMX-157	--	--	--
	MDMX-159	--	--	--
	MDMX-161	chr1:182177575-197158959	chr4:132319029-155629052	--
		chr16:38784309-98280275	chr8:88178406-131708519	--
	Recurrent	--	--	--
**p53TKO**	P53-833	--	--	--
	P53-837	--	--	--
	P53-163	chr3:3272312-8046487	--	--
	P53-165	--	--	--
	P53-167	--	--	--
	P53-169	chr12:10152599-17771536	--	--
		chr12:51257671-58510365		--
	Recurrent	--	--	--

Supplemental Table 7. aCGH recurrent focal lesions

Model	Location	Change	No. Samples	Gene
**MDMX**	chr1:181123492-181345257	Amplification	2	Smyd3
	chr11:3030786-3100864	Amplification	2	Pisd-ps1//Pisd-ps3//Sfi1
	chr11:71030118-71044719	Amplification	3	Nlrp1b
**p53TKO**	chr7:121512739-121546676	Amplification	2	4933406I18Rik
	chr11:71015029-71105158	Amplification	2	Nlrp1b//Nlrp1c-ps
	chr12:116274536-116296062	Amplification	2	
**RbTKO**	chr1:157059324-157083954	Amplification	5	
	chr1:191942355-191994761	Amplification	5	Prox1
	chr10:25710605-25799209	Amplification	5	Gm9767//Tmem200a
	chr10:76224361-76263562	Amplification	5	Pcbp3
	chr10:90609648-90634005	Amplification	5	Tmpo
	chr10:116782083-116803767	Amplification	5	Cpsf6
	chr16:67534450-67621525	Amplification	5	Cadm2
	chr19:4776854-4807777	Amplification	5	Rbm14//Rbm4
	chr19:7499795-7557293	Amplification	5	2700081O15Rik//Rtn3
	chr19:19125889-19185052	Amplification	5	Rorb
	chr19:45823622-45853326	Amplification	5	Mgea5//Npm3
	chr2:6742745-6793948	Amplification	5	Celf2
	chr2:17612655-17647492	Amplification	5	Nebl
	chr2:44867755-44969639	Amplification	5	Gm13476//Zeb2
	chr2:103605511-103637663	Amplification	5	Caprin1
	chr2:155973604-156010448	Amplification	5	Rbm39
	chr5:46108597-46249523	Amplification	5	Gm3414//Lcorl
	chr6:18376392-18455454	Amplification	5	Cttnbp2
	chr7:116280939-116319569	Amplification	5	Lmo1
	chr8:54671964-54692786	Amplification	5	Neil3
	chr8:92841574-92866689	Amplification	5	Tox3
	chr1:52241887-52270415	Amplification	4	Gls
	chr1:155303311-155332457	Amplification	4	Dhx9
	chr10:62212597-62243479	Amplification	4	Ccar1
	chr11:18764949-18904702	Amplification	4	Meis1
	chr11:78425210-78509998	Amplification	4	Nlk
	chr11:102151631-102176210	Amplification	4	Atxn7l3//Ubtf
	chr14:62242846-62267383	Amplification	4	Dleu2//Mir15a//Mir16-1
	chr15:36699224-36721024	Amplification	4	Ywhaz
	chr15:66768886-66785815	Amplification	4	Ndrg1
	chr17:10399809-10452274	Amplification	4	Qk
	chr17:32396649-32448427	Amplification	4	Akap8//Brd4
	chr17:88398947-88411282	Amplification	4	Fbxo11
	chr18:55121633-55148712	Amplification	4	Zfp608
	chr2:11379578-11414175	Amplification	4	Pfkfb3
	chr2:124920396-124943129	Amplification	4	Myef2

chr3:107104574-107135593	Amplification	4	Rbm15//Slc16a4
chr3:119422182-119450153	Amplification	4	Ptbp2
chr4:126600705-126645897	Amplification	4	Zmym4
chr5:132899017-133019656	Amplification	4	Auts2
chr7:142886464-142907661	Amplification	4	Mki67
chr8:48265188-48286266	Amplification	4	Stox2
chr9:88343454-88373663	Amplification	4	Syncrip
chr1:24027529-24106682	Amplification	3	Fam135a
chr1:134461716-134500342	Amplification	3	Nfasc
chr1:134881416-134914488	Amplification	3	Mdm4
chr1:181674593-181719117	Amplification	3	Ahctf1
chr1:183745323-183770600	Amplification	3	Lbr
chr1:193374628-193394752	Amplification	3	Dtl
chr10:80856692-80883315	Amplification	3	2210404O07Rik//Nfic
chr11:74485537-74539602	Amplification	3	Pafah1b1
chr11:88150762-88579817	Amplification	3	Msi2
chr11:98854758-98885007	Amplification	3	Top2a
chr11:102337486-102383440	Amplification	3	Gpatch8
chr11:104210165-104293952	Amplification	3	1700081L11Rik
chr11:113588264-113612923	Amplification	3	Cdc42ep4
chr13:9683712-9759675	Amplification	3	Zmynd11
chr13:64234316-64254484	Amplification	3	Zfp367
chr13:104529015-104703939	Amplification	3	Erbb2ip//Srsf12
chr13:114382445-114408314	Amplification	3	Snx18
chr14:21318616-21339256	Amplification	3	Ppp3cb
chr15:38418463-38449272	Amplification	3	Azin1
chr15:57933295-57958999	Amplification	3	Atad2
chr15:58780254-58912189	Amplification	3	Mtss1
chr15:98368725-98391530	Amplification	3	Ccnt1
chr15:99448426-99480017	Amplification	3	Racgap1
chr15:103021712-103068558	Amplification	3	Cbx5
chr16:11200771-11220806	Amplification	3	Gspt1//Rsl1d1
chr16:18244057-18291074	Amplification	3	Dgcr8//Mir1306//Ranbp1//Trmt2a
chr16:22235745-22265915	Amplification	3	Tra2b
chr16:48952948-48976761	Amplification	3	Dzip3
chr16:91014725-91034938	Amplification	3	4930404I05Rik//Gcfc1
chr16:94967102-95218805	Amplification	3	Kcnj6
chr17:89116959-89191213	Amplification	3	Lhcgr
chr18:21012801-21033009	Amplification	3	Trappc8
chr18:25883724-25910063	Amplification	3	Celf4
chr19:58869314-58935465	Amplification	3	Hspa12a
chr2:72774060-72815198	Amplification	3	Sp3
chr2:80331625-80375037	Amplification	3	Nckap1
chr2:127635014-127649116	Amplification	3	Bub1
chr2:156836570-156843931	Amplification	3	9830001H06Rik
chr2:180756856-180852430	Amplification	3	Chrna4//Kcnq2

chr3:27042849-27064977	Amplification	3	Ect2
chr3:51131870-51152987	Amplification	3	
chr3:51883163-51909325	Amplification	3	Maml3
chr3:95564426-95599369	Amplification	3	Rprd2
chr3:107461092-107499098	Amplification	3	Ahcyl1
chr3:122240729-122265171	Amplification	3	Fnbp1l
chr3:123228855-123302361	Amplification	3	Ndst3
chr4:98601707-98611237	Amplification	3	Dock7//Usp1
chr4:98737900-98785773	Amplification	3	Dock7
chr4:141233985-141241286	Amplification	3	Ddi2//Rsc1a1
chr5:96500513-96591251	Amplification	3	Cnot6l
chr5:135076951-135112510	Amplification	3	Eif4h//Lat2
chr5:151438282-151464838	Amplification	3	N4bp2l2
chr6:47481129-47542995	Amplification	3	Ezh2
chr6:125119960-125143148	Amplification	3	Mrpl51//Ncapd2//Scarna10
chr6:148936412-149049773	Amplification	3	Dennd5b
chr7:17079336-17098529	Amplification	3	Grlf1
chr7:52632102-52651138	Amplification	3	Snrnp70
chr7:74376094-74446474	Amplification	3	Mef2a
chr7:89025827-89078850	Amplification	3	Hdgfrp3//Tm6sf1
chr7:118881283-118923866	Amplification	3	Galntl4
chr8:9539270-9625708	Amplification	3	Fam155a
chr8:10984223-11007745	Amplification	3	Irs2
chr8:23742433-23762458	Amplification	3	A930013F10Rik//Polb
chr9:21819163-21858847	Amplification	3	Elavl3
chr9:59236502-59258886	Amplification	3	Arih1
chr9:82760558-82867378	Amplification	3	Phip
chr9:107642594-107668451	Amplification	3	Rbm5
chr11:3030786-3097537	Deletion	3	Pisd-ps1//Pisd-ps3//Sfi1
chr4:145035178-146907163	Deletion	3	1700029I01Rik//Rex2//Vmn2r-ps14//Zfp600
chr1:9657229-9738482	Amplification	2	Mybl1//Vcpip1
chr1:25705417-25887245	Amplification	2	Bai3
chr1:55041103-55072352	Amplification	2	Sf3b1
chr1:71109091-71143278	Amplification	2	Bard1
chr1:164634739-164666791	Amplification	2	Prrc3
chr1:178663505-178741446	Amplification	2	Cep170//Mir350
chr1:179137923-179176100	Amplification	2	Akt3
chr1:183103185-183133984	Amplification	2	Wdr26
chr10:39359282-39451326	Amplification	2	Traf3ip2
chr10:66470589-66562689	Amplification	2	Reep3
chr10:87547441-87612188	Amplification	2	4930547N16Rik//Nup37//Pmch
chr10:104950470-105011276	Amplification	2	Tmtc2
chr10:126662707-126684908	Amplification	2	Kif5a
chr11:6367838-6375434	Amplification	2	Purb
chr11:40516992-40570069	Amplification	2	Ccng1//Hmmr//Nudcd2
chr11:71031764-71055713	Amplification	2	Nlrp1b

chr11:71077444-71105158	Amplification	2	Nlrp1c-ps
chr11:77342434-77368366	Amplification	2	Taok1
chr11:80906176-80974725	Amplification	2	Accn1
chr11:97020558-97201383	Amplification	2	Gpr179//Kpnb1//Mrpl45//Npepps
chr11:100290040-100326433	Amplification	2	Klhl10//Klhl11//Nt5c3l
chr11:102060979-102091004	Amplification	2	Hdac5
chr12:110270302-110298129	Amplification	2	Begain
chr13:54780356-54866817	Amplification	2	Cdhr2//Gprin1//Rnf44//Sncb
chr13:56174425-56200124	Amplification	2	H2afy
chr13:59601288-59631668	Amplification	2	Agtpbp1
chr13:69633810-69673280	Amplification	2	Papd7
chr13:77210096-77288747	Amplification	2	2210408I21Rik//Ankrd32
chr14:30468675-30533934	Amplification	2	Cacna2d3
chr14:57338677-57386280	Amplification	2	Pspc1
chr15:9041755-9068886	Amplification	2	Skp2
chr15:25309088-25343873	Amplification	2	Basp1
chr15:27901877-27951605	Amplification	2	Trio
chr15:37914920-38006976	Amplification	2	Ubr5
chr15:51793682-51824737	Amplification	2	Rad21
chr15:68167018-68208130	Amplification	2	Mir30b//Mir30d
chr15:76001639-76022194	Amplification	2	Plec
chr15:76907143-76916702	Amplification	2	Rbfox2
chr15:79358603-79435635	Amplification	2	Ddx17//Dmc1
chr15:81534339-81555309	Amplification	2	Rangap1
chr15:98640270-98685166	Amplification	2	Mll2//Prkag1
chr16:42811912-42836975	Amplification	2	
chr17:24204344-24227641	Amplification	2	Kctd5//Pdpk1
chr17:29107535-29157894	Amplification	2	Stk38
chr17:32903496-32946643	Amplification	2	Zfp811//Zfp871
chr17:33783472-33813955	Amplification	2	Hnrnpm
chr17:56550443-56588596	Amplification	2	Ptprs
chr17:66734051-66805414	Amplification	2	1110012J17Rik
chr18:16852875-16926705	Amplification	2	Cdh2
chr18:31883059-31906673	Amplification	2	
chr18:46652963-46676495	Amplification	2	Fem1c
chr18:64373035-64422823	Amplification	2	St8sia3
chr18:79255160-79283224	Amplification	2	Setbp1
chr19:3503263-3527696	Amplification	2	Ppp6r3
chr19:8866353-8894993	Amplification	2	Hnrnpul2//Polr2g//Ttc9c//Zbtb3
chr19:9935299-9968228	Amplification	2	Incenp
chr19:23281881-23305890	Amplification	2	Smc5
chr19:28029792-28086554	Amplification	2	Rfx3
chr19:41676553-41818250	Amplification	2	Slit1
chr19:47065211-47082866	Amplification	2	Nt5c2
chr2:22961252-22983156	Amplification	2	Acbd5//Mastl
chr2:48772032-48796446	Amplification	2	Orc4

chr2:113649373-113688606	Amplification	2	Arhgap11a//Scg5
chr2:120555735-120610958	Amplification	2	Cdan1//Ttbk2
chr2:129093478-129121054	Amplification	2	Ckap2l
chr2:130531488-130655034	Amplification	2	4930402H24Rik//Gm14057
chr2:134420046-134457578	Amplification	2	Tmx4
chr2:144075071-144096412	Amplification	2	Snord17//Snx5
chr2:168005844-168041258	Amplification	2	Adnp//Dpm1
chr2:173046994-173101982	Amplification	2	Pmepa1
chr3:17167694-17231410	Amplification	2	
chr3:126644772-126864980	Amplification	2	Ank2
chr4:34751024-34829138	Amplification	2	Zfp292
chr4:41194856-41218322	Amplification	2	Ubap2
chr4:44138184-44177039	Amplification	2	Rnf38
chr4:83098915-83130525	Amplification	2	Psip1//Snapc3
chr4:111989244-112064307	Amplification	2	Skint9
chr4:116273328-116283798	Amplification	2	Nasp
chr4:116832720-116884147	Amplification	2	Kif2c
chr4:123025959-123364615	Amplification	2	D830031N03Rik//Macf1
chr4:133235493-133309190	Amplification	2	Arid1a
chr4:134356593-134410065	Amplification	2	Tmem57
chr4:134995295-135019655	Amplification	2	Nipal3
chr5:22917209-22941152	Amplification	2	Mll5
chr5:24308896-24349072	Amplification	2	Rheb
chr5:36943148-37070223	Amplification	2	D5Ertd579e
chr5:64495741-64521497	Amplification	2	Pgm1
chr5:65649539-65736551	Amplification	2	Rfc1//Wdr19
chr5:86437901-86492358	Amplification	2	Cenpc1
chr5:100385370-100405698	Amplification	2	Hnrnpd
chr5:107055215-107126127	Amplification	2	Zfp644
chr5:116891245-116917756	Amplification	2	2410137F16Rik//Srrm4
chr5:131971167-132009354	Amplification	2	Auts2
chr5:132103764-132246968	Amplification	2	Auts2
chr5:132326694-132504802	Amplification	2	Auts2
chr6:12579116-12699178	Amplification	2	Thsd7a
chr6:27351676-27375621	Amplification	2	Grm8
chr6:28763693-28783820	Amplification	2	Lrrc4//Snd1
chr6:35055697-35084173	Amplification	2	Cnot4
chr6:42093811-42131498	Amplification	2	
chr6:112547047-112614236	Amplification	2	Rad18
chr6:121322490-121376322	Amplification	2	Iqsec3
chr6:143010913-143034041	Amplification	2	5730419I09Rik
chr7:16913133-16972865	Amplification	2	Sae1
chr7:31017685-31041668	Amplification	2	Polr2i//Wdr62
chr7:62891780-62925649	Amplification	2	
chr7:72573719-72659688	Amplification	2	
chr7:80662972-80698872	Amplification	2	1810026B05Rik//Chd2

chr7:117071488-117102353	Amplification	2	Dennd5a
chr7:126278212-126328097	Amplification	2	Gpr139
chr7:135583817-135604361	Amplification	2	Tial1
chr7:140193111-140314652	Amplification	2	Ctbp2
chr7:150401535-150481152	Amplification	2	Kcnq1//Kcnq1ot1
chr8:4737623-4765494	Amplification	2	Shcbp1
chr8:27131409-27145998	Amplification	2	Hook3
chr8:33491459-33994687	Amplification	2	
chr9:4606601-4795357	Amplification	2	Gria4
chr9:15124414-15140109	Amplification	2	5830418K08Rik//Scarna9
chr9:19977294-20011623	Amplification	2	
chr9:20722306-20754876	Amplification	2	Dnmt1
chr9:49309654-49607067	Amplification	2	Gm11149//Ncam1
chr9:62640145-62830124	Amplification	2	Calml4//Cln6//Fem1b//Gm10653//Pias1
chr9:66363730-66440674	Amplification	2	Usp3
chr9:89985540-90021888	Amplification	2	Morf4l1
chr9:106968565-107061268	Amplification	2	Dock3

Supplemental Table 8. Exome sequencing single nucleotide variations (SNV)

Sample	chr	pos	#Mut_in_Tumor	#Total_in_Tumor	#Mut_in_normal	#Total_in_normal	Ref_Allele	Mut_Allele	p_val	Type	gene	variant_class	variant_change
SJRB010140	chr6	149460634	80	265	1	322	A	C	5.53E-30	Somatic	Bicd1	missense	I334L.M334L_chr12:32350318
SJRB010140	chr13	101481476	246	657	2	610	A	G	1.47E-78	Somatic	Cdk7	silent	C201C.C201C_chr5:68593863
SJRB010140	chr7	26323764	136	412	2	410	C	T	1.21E-43	Somatic	Ceacam2	missense	G85S.D85N_chr19:46775580
SJRB010140	chr1	174628313	191	626	0	726	C	T	1.41E-72	Somatic	Crp	missense	R16W.H15Y_chr1:157950857
SJRB010140	chr6	38141752	150	444	0	452	C	T	3.88E-53	Somatic	D630045J12Rik	missense	R954Q.R964K_chr7:138247734
SJRB010140	chr8	95441991	113	288	1	386	A	G	2.45E-47	Somatic	Lpcat2	missense	Y503C.Y503C_chr16:54174384
SJRB010140	chr2	85824701	180	488	1	593	C	T	1.80E-70	Somatic	Olfr1030	missense	R235C.NA_NA
SJRB010140	chr4	62362073	68	173	1	176	T	A	2.06E-23	Somatic	Rgs3	missense	L852I.L405I,L803I,L1084I,L54I,L197I_chr9:115397705
SJRB010140	chr5	73403214	30	83	0	138	G	C	3.38E-15	Somatic	Slc10a4	silent	S314S.S314S_chr4:48185341
SJRB010140	chr6	113654793	38	97	1	175	G	T	9.42E-19	Somatic	Tatdn2	nonsense	E537*.E576*_chr3:10287592
SJRB010140	chr1	21003482	126	382	0	378	C	T	1.18E-43	Somatic	Tram2	splice	D98_E3splice.NA_NA
SJRB010145	chr6	91869226	20	558	0	523	C	T	1.93E-06	Somatic	4930590J08Rik	nonsense	Q354*.H358Y_NA
SJRB010146	chr14	32065142	44	410	0	312	T	C	6.05E-12	Somatic	Bap1	splice	G41_E3splice.NA_NA
SJRB010146	chr17	29828357	158	599	2	491	G	A	1.15E-42	Somatic	Ftsjd2	missense	R497H.R498Q_chr6:37538750
SJRB010146	chr1	132807865	92	1117	0	682	T	C	2.30E-20	Somatic	Il20	UTR_5	E1_UTR_5.NA_NA
SJRB010146	chr13	72768205	21	205	0	122	G	A	6.86E-05	Somatic	Irx2	missense	E147K.E146K_chr5:2802715
SJRB010146	chr13	67887813	81	868	0	698	G	A	3.20E-22	Somatic	Zfp493	missense	C316Y.NA_NA
SJRB010147	chr17	35233465	65	302	0	247	C	G	4.36E-19	Somatic	Abhd16a	missense	A181G.NA_NA
SJRB010147	chr8	126004266	132	379	0	385	C	A	5.37E-47	Somatic	Afg3l1	nonsense	C52*.NA_NA
SJRB010147	chr11	69291587	105	287	0	273	G	A	4.37E-36	Somatic	Dnahc2	silent	R1664R.H1629H_chr17:7620132
SJRB010147	chrX	61404728	75	242	0	176	C	A	5.22E-21	Somatic	Gm6760	UTR_3	E2_UTR_3.NA_NA
SJRB010147	chr12	73170742	116	303	2	338	C	G	2.83E-41	Somatic	Gpr135	missense	A413P.A450P_chr14:59000350
SJRB010147	chrX	7389985	151	421	2	172	T	C	3.69E-24	Somatic	Gripap1	silent	L585L.L620L,L541L_chrX:48722643
SJRB010147	chr2	84797981	105	519	0	370	C	G	3.60E-27	Somatic	Slc43a3	UTR_3	E13_UTR_3.NA_chr11:56931791
SJRB010147	chr16	17640315	109	337	5	311	T	C	7.10E-29	Somatic	Smpd4	silent	N461N.NA_NA
SJRB010147	chr1	74734779	57	262	1	289	A	G	3.78E-19	Somatic	Ttll4	missense	H802R.H807R_chr2:219321113
SJRB010148	chr7	130465173	196	897	1	652	C	T	1.25E-49	Somatic	Arhgap17	silent	E130E.E130E_chr16:24887244
SJRB010148	chr8	23488450	77	602	2	564	A	G	6.24E-21	Somatic	Slc25a15	missense	I262T.I262T_chr13:40281682
SJRB010148	chr19	8816850	27	228	1	285	A	G	2.11E-09	Somatic	Stx5a	missense	T30A.T30A_chr11:62355204
SJRB010149	chr7	13476431	145	428	0	498	G	A	3.42E-56	Somatic	Zfp128	missense	R459Q.R462Q_chr19:63498371
SJRB010150	chr5	141354018	31	274	0	392	C	T	3.84E-13	Somatic	Card11	missense	R973H.R973H_chr7:2919548
SJRB010150	chr13	100914505	18	234	1	251	T	C	1.77E-05	Somatic	Naip2	missense	I1385V.NA_NA
SJRB010150	chr2	148523913	38	394	1	553	A	G	2.73E-14	Somatic	Napb	splice_region	A262_E10splice_region.NA_NA
SJRB010150	chr12	112032438	85	600	1	629	G	T	1.07E-26	Somatic	Wdr20a	missense	R516L.NA_NA

Supplemental Table 9. Exome sequencing insertions and deletions

Sample	Chr	GeneName	mm9_Pos	Class	AAChange	ProteinGI	mRNA_acc	#Mutant In Tumor	#Total In Tumor	#Mutant In Normal	#Total In Normal	ReferenceAllele	MutantAllele	Flanking	Validation
SJRB010144	chr8	Tmco7	109266076	proteinDel	Q768_L771>L	27370560	NM_173037	4	147	0	141	AGAGTACCC	-	ACAGCAGCCC(AGAGTACCC>---------)TAAACCAAAA	somatic
SJRB010147	chr7	Gde1	125842064	splice_region	L145_E2splice_region	9625018	NM_019580	12	23	0	26	-	A	GATGTCAGGG(->A)CTCACCTGAG	somatic
SJRB010150	chr10	Bicc1	70394957	frameshift	C859fs	13994223	NM_031397	3	37	0	21	-	G	CGAGGAAATG(->G)CAGTCCATGT	somatic

Supplemental Table 10: Mouse retinoblastoma integrative data analysis

Symbol	Annotation	Empirical p value	FDR adjusted empirical p value	Cancer Gene
Tk1	thymidine kinase 1	2.0E-06	3.3E-03	N
Galntl4	UDP-N-acetyl-alpha-D-galactosamine:polypeptide N-acetylgalactosaminyltransferase-like 4	2.8E-05	1.1E-02	N
Rfc4	replication factor C (activator 1) 4	3.6E-05	1.2E-02	N
B3galt5	UDP-Gal:betaGlcNAc beta 1,3-galactosyltransferase, polypeptide 5	4.4E-05	1.3E-02	N
Rad18	RAD18 homolog (S. cerevisiae)	7.8E-05	1.6E-02	N
Akr1b3	aldo-keto reductase family 1, member B3 (aldose reductase)	1.1E-04	2.1E-02	N
Kcnj9	potassium inwardly-rectifying channel, subfamily J, member 9	1.2E-04	2.1E-02	N
Pole	polymerase (DNA directed), epsilon	2.4E-04	3.5E-02	N
Cdca7l	cell division cycle associated 7 like	2.7E-04	3.5E-02	N
Fbxo5	F-box protein 5	2.7E-04	3.5E-02	N
Otoa	otoancorin	2.8E-04	3.5E-02	N
Wee1	WEE 1 homolog 1 (S. pombe)	3.5E-04	3.8E-02	N
Sgk3	serum/glucocorticoid regulated kinase 3	3.5E-04	3.8E-02	N
Kntc1	kinetochore associated 1	3.7E-04	3.8E-02	N
Drd2	dopamine receptor D2	4.9E-04	4.6E-02	N
Rrm1	ribonucleotide reductase M1	5.7E-04	4.9E-02	N
Serpinb9	serine (or cysteine) peptidase inhibitor, clade B, member 9	6.2E-04	5.0E-02	N
Neurod6	neurogenic differentiation 6	6.3E-04	5.0E-02	N
Fah	fumarylacetoacetate hydrolase	6.3E-04	5.0E-02	N
Tpm3	tropomyosin 3, gamma	7.2E-04	5.5E-02	Y
Uhrf1	ubiquitin-like, containing PHD and RING finger domains, 1	7.6E-04	5.5E-02	N
Cdkn1a	cyclin-dependent kinase inhibitor 1A (P21)	7.6E-04	5.5E-02	N
Gdpd3	glycerophosphodiester phosphodiesterase domain containing 3	7.7E-04	5.5E-02	N
Cdkn2c	cyclin-dependent kinase inhibitor 2C (p18, inhibits CDK4)	8.1E-04	5.6E-02	Y
Rnase4	ribonuclease, RNase A family 4	9.5E-04	6.3E-02	N
Shcbp1	Shc SH2-domain binding protein 1	9.7E-04	6.3E-02	N
Kctd9	potassium channel tetramerisation domain containing 9	1.0E-03	6.4E-02	N
St8sia5	ST8 alpha-N-acetyl-neuraminide alpha-2,8-sialyltransferase 5	1.1E-03	6.4E-02	N
Hells	helicase, lymphoid specific	1.1E-03	6.7E-02	N
Lnp	limb and neural patterns	1.3E-03	6.9E-02	N
Nek2	NIMA (never in mitosis gene a)-related expressed kinase 2	1.3E-03	6.9E-02	N
Lmo4	LIM domain only 4	1.4E-03	7.5E-02	N
Dtl	denticleless homolog (Drosophila)	1.5E-03	7.8E-02	N
Fgf1	fibroblast growth factor 1	1.6E-03	8.3E-02	N
Sgpp1	sphingosine-1-phosphate phosphatase 1	1.6E-03	8.3E-02	N
Mcm8	minichromosome maintenance deficient 8 (S. cerevisiae)	2.0E-03	9.8E-02	N
Cdr2	cerebellar degeneration-related 2	2.1E-03	1.0E-01	N
Stk35	serine/threonine kinase 35	2.2E-03	1.0E-01	N
Cdkn2a	cyclin-dependent kinase inhibitor 2A	2.2E-03	1.0E-01	Y
Lbr	lamin B receptor	2.4E-03	1.1E-01	N
Msh6	mutS homolog 6 (E. coli)	2.5E-03	1.1E-01	Y
D10627	cDNA sequence D10627	2.5E-03	1.1E-01	N
Bola2	bolA-like 2 (E. coli)	2.5E-03	1.1E-01	N
Zfpm2	zinc finger protein, multitype 2	2.5E-03	1.1E-01	N
Phf19	PHD finger protein 19	2.5E-03	1.1E-01	N
Actc1	actin, alpha, cardiac muscle 1	2.6E-03	1.1E-01	N
Topbp1	topoisomerase (DNA) II binding protein 1	2.7E-03	1.1E-01	N
Bub1b	budding uninhibited by benzimidazoles 1 homolog, beta (S. cerevisiae)	2.9E-03	1.1E-01	Y
Usp1	ubiquitin specific peptidase 1	2.9E-03	1.1E-01	N
Cbx5	chromobox homolog 5 (Drosophila HP1a)	3.1E-03	1.1E-01	N
2610020H08Rik	RIKEN cDNA 2610020H08 gene	3.1E-03	1.1E-01	N
Usp2	ubiquitin specific peptidase 2	3.2E-03	1.1E-01	N
Col27a1	collagen, type XXVII, alpha 1	3.2E-03	1.1E-01	N
Lrba	LPS-responsive beige-like anchor	3.2E-03	1.2E-01	N
Cit	citron	3.6E-03	1.2E-01	N
Nudt6	nudix (nucleoside diphosphate linked moiety X)-type motif 6	3.6E-03	1.2E-01	N
Cobll1	Cobl-like 1	3.7E-03	1.2E-01	N
Camkv	CaM kinase-like vesicle-associated	3.7E-03	1.2E-01	N
Tmpo	thymopoietin	3.9E-03	1.3E-01	N
Ube2t	ubiquitin-conjugating enzyme E2T (putative)	4.1E-03	1.3E-01	N
Npas4	neuronal PAS domain protein 4	4.4E-03	1.3E-01	N
Pmf1	polyamine-modulated factor 1	4.4E-03	1.3E-01	N
Rbp7	retinol binding protein 7, cellular	4.4E-03	1.3E-01	N
Pold1	polymerase (DNA directed), delta 1, catalytic subunit	4.6E-03	1.4E-01	N
Ifi47	interferon gamma inducible protein 47	4.8E-03	1.4E-01	N

Pola1	polymerase (DNA directed), alpha 1	5.1E-03	1.5E-01	N
Fxyd6	FXYD domain-containing ion transport regulator 6	5.3E-03	1.5E-01	N
Ube2c	ubiquitin-conjugating enzyme E2C	5.3E-03	1.5E-01	N
Man1a	mannosidase 1, alpha	5.4E-03	1.5E-01	N
Pim1	proviral integration site 1	5.5E-03	1.5E-01	Y
Mad2l1	MAD2 mitotic arrest deficient-like 1 (yeast)	5.5E-03	1.5E-01	N
Clspn	claspin homolog (Xenopus laevis)	5.6E-03	1.5E-01	N
Arhgap19	Rho GTPase activating protein 19	5.6E-03	1.5E-01	N
Cep55	centrosomal protein 55	5.7E-03	1.5E-01	N
Mcm2	minichromosome maintenance deficient 2 mitotin (S. cerevisiae)	5.9E-03	1.5E-01	N
Ctps	cytidine 5'-triphosphate synthase	6.0E-03	1.5E-01	N
D2Ertd750e	DNA segment, Chr 2, ERATO Doi 750, expressed	6.3E-03	1.6E-01	N
Trim59	tripartite motif-containing 59	6.3E-03	1.6E-01	N
Nxt1	NTF2-related export protein 1	6.3E-03	1.6E-01	N
C230052I12Rik	RIKEN cDNA C230052I12 gene	6.4E-03	1.6E-01	N
1810035L17Rik	RIKEN cDNA 1810035L17 gene	6.6E-03	1.6E-01	N
Rbmx2	RNA binding motif protein, X-linked 2	6.8E-03	1.6E-01	N
Zbtb8os	zinc finger and BTB domain containing 8 opposite strand	6.8E-03	1.6E-01	N
Aldh1l2	aldehyde dehydrogenase 1 family, member L2	7.0E-03	1.6E-01	N
Atf5	activating transcription factor 5	7.1E-03	1.6E-01	N
Sdk2	sidekick homolog 2 (chicken)	7.3E-03	1.6E-01	N
Prkar2b	protein kinase, cAMP dependent regulatory, type II beta	7.4E-03	1.6E-01	N
Nup50	nucleoporin 50	7.5E-03	1.6E-01	N
Scd1	stearoyl-Coenzyme A desaturase 1	7.6E-03	1.6E-01	N
Bsnd	Bartter syndrome, infantile, with sensorineural deafness (Barttin)	8.1E-03	1.7E-01	N
Id2	inhibitor of DNA binding 2	8.3E-03	1.7E-01	N
Xkr5	X Kell blood group precursor-related family, member 5	8.7E-03	1.7E-01	N
6720463M24Rik	RIKEN cDNA 6720463M24 gene	8.9E-03	1.8E-01	N
Trpc2	transient receptor potential cation channel, subfamily C, member 2	9.1E-03	1.8E-01	N
Nubp1	nucleotide binding protein 1	9.3E-03	1.8E-01	N
Cdca5	cell division cycle associated 5	9.3E-03	1.8E-01	N
Lrdd	leucine-rich and death domain containing	9.9E-03	1.9E-01	N
Utrn	utrophin	1.0E-02	1.9E-01	N
Ddx11	DEAD/H (Asp-Glu-Ala-Asp/His) box polypeptide 11 (CHL1-like helicase homolog, S. cerevisiae)	1.0E-02	1.9E-01	N
Drd4	dopamine receptor D4	1.1E-02	1.9E-01	N
Mdc1	mediator of DNA damage checkpoint 1	1.1E-02	1.9E-01	N
Neil3	nei like 3 (E. coli)	1.1E-02	1.9E-01	N
Celsr3	cadherin, EGF LAG seven-pass G-type receptor 3 (flamingo homolog, Drosophila)	1.1E-02	1.9E-01	N
Rell1	RELT-like 1	1.2E-02	2.0E-01	N
Ccnb2	cyclin B2	1.3E-02	2.1E-01	N
Rad51c	RAD51 homolog c (S. cerevisiae)	1.3E-02	2.1E-01	N
Hdac9	histone deacetylase 9	1.3E-02	2.1E-01	N
Prim1	DNA primase, p49 subunit	1.3E-02	2.1E-01	N
Stat1	signal transducer and activator of transcription 1	1.3E-02	2.1E-01	N
Ing2	inhibitor of growth family, member 2	1.3E-02	2.1E-01	N
Itgb3bp	integrin beta 3 binding protein (beta3-endonexin)	1.3E-02	2.1E-01	N
Thoc4	THO complex 4	1.3E-02	2.1E-01	N
Plk1	polo-like kinase 1 (Drosophila)	1.4E-02	2.1E-01	N
E330016A19Rik	RIKEN cDNA E330016A19 gene	1.4E-02	2.1E-01	N
Slc7a3	solute carrier family 7 (cationic amino acid transporter, y+ system), member 3	1.4E-02	2.1E-01	N
Adamts4	a disintegrin-like and metallopeptidase (reprolysin type) with thrombospondin type 1 mo	1.4E-02	2.1E-01	N
Kif4	kinesin family member 4	1.4E-02	2.1E-01	N
Leprel1	leprecan-like 1	1.4E-02	2.1E-01	N
Lrrc4	leucine rich repeat containing 4	1.4E-02	2.1E-01	N
2810417H13Rik	RIKEN cDNA 2810417H13 gene	1.4E-02	2.1E-01	N
Apoe	apolipoprotein E	1.4E-02	2.1E-01	N
Cdc42ep4	CDC42 effector protein (Rho GTPase binding) 4	1.5E-02	2.1E-01	N
Hspb3	heat shock protein 3	1.5E-02	2.1E-01	N
Cep76	centrosomal protein 76	1.5E-02	2.1E-01	N
Gna14	guanine nucleotide binding protein, alpha 14	1.6E-02	2.2E-01	N
Rassf2	Ras association (RalGDS/AF-6) domain family member 2	1.6E-02	2.2E-01	N
Cxcl10	chemokine (C-X-C motif) ligand 10	1.7E-02	2.2E-01	N
Scml2	sex comb on midleg-like 2 (Drosophila)	1.7E-02	2.2E-01	N
Adarb2	adenosine deaminase, RNA-specific, B2	1.7E-02	2.2E-01	N
Mmp12	matrix metallopeptidase 12	1.7E-02	2.2E-01	N
Aldh1b1	aldehyde dehydrogenase 1 family, member B1	1.7E-02	2.2E-01	N
Anln	anillin, actin binding protein	1.7E-02	2.2E-01	N
Ube2s	ubiquitin-conjugating enzyme E2S	1.7E-02	2.2E-01	N
Trip13	thyroid hormone receptor interactor 13	1.8E-02	2.3E-01	N

Lrrc8c	leucine rich repeat containing 8 family, member C	1.8E-02	2.3E-01	N
F630043A04Rik	RIKEN cDNA F630043A04 gene	1.9E-02	2.3E-01	N
Nup62	nucleoporin 62	1.9E-02	2.3E-01	N
Dtnbp1	dystrobrevin binding protein 1	2.0E-02	2.3E-01	N
2610021K21Rik	RIKEN cDNA 2610021K21 gene	2.0E-02	2.3E-01	N
Rad50	RAD50 homolog (S. cerevisiae)	2.0E-02	2.3E-01	N
Birc5	baculoviral IAP repeat-containing 5	2.0E-02	2.3E-01	N
Rras	Harvey rat sarcoma oncogene, subgroup R	2.1E-02	2.3E-01	N
Gen1	Gen homolog 1, endonuclease (Drosophila)	2.2E-02	2.4E-01	N
Phlda3	pleckstrin homology-like domain, family A, member 3	2.2E-02	2.4E-01	N
4930422G04Rik	RIKEN cDNA 4930422G04 gene	2.2E-02	2.4E-01	N
Il23a	interleukin 23, alpha subunit p19	2.2E-02	2.4E-01	N
Ap1s2	adaptor-related protein complex 1, sigma 2 subunit	2.2E-02	2.4E-01	N
Mxd3	Max dimerization protein 3	2.3E-02	2.4E-01	N
Aspm	asp (abnormal spindle)-like, microcephaly associated (Drosophila)	2.3E-02	2.4E-01	N
Tcf19	transcription factor 19	2.4E-02	2.5E-01	N
Rap1b	RAS related protein 1b	2.4E-02	2.5E-01	N
Tmie	transmembrane inner ear	2.4E-02	2.5E-01	N
Ctsz	cathepsin Z	2.5E-02	2.5E-01	N
Tspan4	tetraspanin 4	2.5E-02	2.5E-01	N
Zmat3	zinc finger matrin type 3	2.5E-02	2.5E-01	N
Rhobtb1	Rho-related BTB domain containing 1	2.5E-02	2.5E-01	N
Slc1a7	solute carrier family 1 (glutamate transporter), member 7	2.5E-02	2.5E-01	N
Plce1	phospholipase C, epsilon 1	2.4E-02	2.5E-01	N
Pdzd2	PDZ domain containing 2	2.4E-02	2.4E-01	N
BC027072	cDNA sequence BC027072	2.4E-02	2.4E-01	N
Tdrd9	tudor domain containing 9	2.4E-02	2.4E-01	N
Chst15	carbohydrate (N-acetylgalactosamine 4-sulfate 6-O) sulfotransferase 15	2.3E-02	2.4E-01	N
Meis2	Meis homeobox 2	2.3E-02	2.4E-01	N
Mpp4	membrane protein, palmitoylated 4 (MAGUK p55 subfamily member 4)	2.3E-02	2.4E-01	N
Tac1	tachykinin 1	2.3E-02	2.4E-01	N
Gnb3	guanine nucleotide binding protein (G protein), beta 3	2.3E-02	2.4E-01	N
Moxd1	monooxygenase, DBH-like 1	2.3E-02	2.4E-01	N
Fabp12	fatty acid binding protein 12	2.2E-02	2.4E-01	N
Ngfr	nerve growth factor receptor (TNFR superfamily, member 16)	2.2E-02	2.4E-01	N
Kcnab1	potassium voltage-gated channel, shaker-related subfamily, beta member 1	2.2E-02	2.4E-01	N
Crx	cone-rod homeobox containing gene	2.2E-02	2.4E-01	N
Tcf7l2	transcription factor 7-like 2, T-cell specific, HMG-box	2.2E-02	2.4E-01	Y
Dock9	dedicator of cytokinesis 9	2.2E-02	2.4E-01	N
Crabp1	cellular retinoic acid binding protein I	2.2E-02	2.4E-01	N
Gpr153	G protein-coupled receptor 153	2.2E-02	2.4E-01	N
Parp8	poly (ADP-ribose) polymerase family, member 8	2.2E-02	2.4E-01	N
Tmprss5	transmembrane protease, serine 5 (spinesin)	2.1E-02	2.3E-01	N
Epha8	Eph receptor A8	2.1E-02	2.3E-01	N
Shroom3	shroom family member 3	2.1E-02	2.3E-01	N
Sdc2	syndecan 2	2.0E-02	2.3E-01	N
Serpinh1	serine (or cysteine) peptidase inhibitor, clade H, member 1	2.0E-02	2.3E-01	N
Fam19a3	family with sequence similarity 19, member A3	2.0E-02	2.3E-01	N
C1ql3	C1q-like 3	2.0E-02	2.3E-01	N
Rpgrip1	retinitis pigmentosa GTPase regulator interacting protein 1	2.0E-02	2.3E-01	N
Snca	synuclein, alpha	2.0E-02	2.3E-01	N
Cplx2	complexin 2	2.0E-02	2.3E-01	N
Dmd	dystrophin, muscular dystrophy	2.0E-02	2.3E-01	N
St14	suppression of tumorigenicity 14 (colon carcinoma)	2.0E-02	2.3E-01	N
Pde6h	phosphodiesterase 6H, cGMP-specific, cone, gamma	2.0E-02	2.3E-01	N
Tgfa	transforming growth factor alpha	1.9E-02	2.3E-01	N
Shh	sonic hedgehog	1.9E-02	2.3E-01	N
Prr5l	proline rich 5 like	1.9E-02	2.3E-01	N
Rax	retina and anterior neural fold homeobox	1.9E-02	2.3E-01	N
Gnat2	guanine nucleotide binding protein, alpha transducing 2	1.9E-02	2.3E-01	N
Ednrb	endothelin receptor type B	1.9E-02	2.3E-01	N
Scube3	signal peptide, CUB domain, EGF-like 3	1.9E-02	2.3E-01	N
Elfn1	leucine rich repeat and fibronectin type III, extracellular 1	1.9E-02	2.3E-01	N
Snhg11	small nucleolar RNA host gene 11	1.9E-02	2.3E-01	N
Tmem178	transmembrane protein 178	1.9E-02	2.3E-01	N
Ece1	endothelin converting enzyme 1	1.8E-02	2.3E-01	N
AI987944	expressed sequence AI987944 /// expressed sequence AW146154	1.8E-02	2.2E-01	N
Nr2e1	nuclear receptor subfamily 2, group E, member 1	1.8E-02	2.2E-01	N
Gldc	glycine decarboxylase	1.7E-02	2.2E-01	N

Cdhr1	cadherin-related family member 1	1.7E-02	2.2E-01	N
Pde6g	phosphodiesterase 6G, cGMP-specific, rod, gamma	1.7E-02	2.2E-01	N
Cntn1	contactin 1	1.7E-02	2.2E-01	N
Flrt2	fibronectin leucine rich transmembrane protein 2	1.7E-02	2.2E-01	N
Enho	energy homeostasis associated	1.7E-02	2.2E-01	N
Col9a3	collagen, type IX, alpha 3	1.7E-02	2.2E-01	N
Nrn1	neuritin 1	1.7E-02	2.2E-01	N
Lrrtm1	leucine rich repeat transmembrane neuronal 1	1.7E-02	2.2E-01	N
Flcn	folliculin	1.6E-02	2.2E-01	N
Sema5a	sema domain, seven thrombospondin repeats (type 1 and type 1-like), transmembrane domain	1.6E-02	2.2E-01	N
Cdh7	cadherin 7, type 2	1.6E-02	2.1E-01	N
BC023829	cDNA sequence BC023829	1.6E-02	2.1E-01	N
Lifr	leukemia inhibitory factor receptor	1.6E-02	2.1E-01	Y
Gulo	gulonolactone (L-) oxidase	1.5E-02	2.1E-01	N
Ccdc113	coiled-coil domain containing 113	1.5E-02	2.1E-01	N
Gpr98	G protein-coupled receptor 98	1.5E-02	2.1E-01	N
Sv2b	synaptic vesicle glycoprotein 2 b	1.5E-02	2.1E-01	N
Gria1	glutamate receptor, ionotropic, AMPA1 (alpha 1)	1.5E-02	2.1E-01	N
Cngb3	cyclic nucleotide gated channel beta 3	1.5E-02	2.1E-01	N
Pex5l	peroxisomal biogenesis factor 5-like	1.5E-02	2.1E-01	N
Opn1sw	opsin 1 (cone pigments), short-wave-sensitive (color blindness, tritan)	1.4E-02	2.1E-01	N
3110007F17Rik	RIKEN cDNA 3110007F17 gene	1.4E-02	2.1E-01	N
Hs3st3b1	heparan sulfate (glucosamine) 3-O-sulfotransferase 3B1	1.4E-02	2.1E-01	N
Zfp385b	zinc finger protein 385B	1.4E-02	2.1E-01	N
Rrh	retinal pigment epithelium derived rhodopsin homolog	1.4E-02	2.1E-01	N
Cckbr	cholecystokinin B receptor	1.4E-02	2.1E-01	N
Rreb1	ras responsive element binding protein 1	1.4E-02	2.1E-01	N
Pcdh11x	protocadherin 11 X-linked	1.3E-02	2.1E-01	N
Cntn4	contactin 4	1.3E-02	2.1E-01	N
Fzd5	frizzled homolog 5 (Drosophila)	1.3E-02	2.1E-01	N
Kcnip1	Kv channel-interacting protein 1	1.3E-02	2.1E-01	N
Gabrb2	gamma-aminobutyric acid (GABA) A receptor, subunit beta 2	1.2E-02	2.0E-01	N
Ass1	argininosuccinate synthetase 1	1.2E-02	2.0E-01	N
Dlc1	deleted in liver cancer 1	1.2E-02	2.0E-01	N
Mtap1b	microtubule-associated protein 1B	1.1E-02	1.9E-01	N
2900026A02Rik	RIKEN cDNA 2900026A02 gene	1.1E-02	1.9E-01	N
Cpne8	copine VIII	1.1E-02	1.9E-01	N
Sat1	spermidine/spermine N1-acetyl transferase 1	1.1E-02	1.9E-01	N
Chrna5	cholinergic receptor, nicotinic, alpha polypeptide 5	1.1E-02	1.9E-01	N
Rgs8	regulator of G-protein signaling 8	1.1E-02	1.9E-01	N
Speg	SPEG complex locus	1.1E-02	1.9E-01	N
D330012F22Rik	RIKEN cDNA D330012F22 gene	1.1E-02	1.9E-01	N
Lrfn5	leucine rich repeat and fibronectin type III domain containing 5	1.1E-02	1.9E-01	N
Abca4	ATP-binding cassette, sub-family A (ABC1), member 4	1.1E-02	1.9E-01	N
Fam132a	family with sequence similarity 132, member A	1.1E-02	1.9E-01	N
Fam53b	family with sequence similarity 53, member B	1.0E-02	1.9E-01	N
Kcna5	potassium voltage-gated channel, shaker-related subfamily, member 5	1.0E-02	1.9E-01	N
Rlbp1	retinaldehyde binding protein 1	9.9E-03	1.9E-01	N
Unc119	unc-119 homolog (C. elegans)	9.7E-03	1.8E-01	N
Galnt4	UDP-N-acetyl-alpha-D-galactosamine:polypeptide N-acetylgalactosaminyltransferase 4	9.2E-03	1.8E-01	N
Slco4a1	solute carrier organic anion transporter family, member 4a1	9.1E-03	1.8E-01	N
Galnt13	UDP-N-acetyl-alpha-D-galactosamine:polypeptide N-acetylgalactosaminyltransferase 13	9.1E-03	1.8E-01	N
Cnp	2',3'-cyclic nucleotide 3' phosphodiesterase	9.1E-03	1.8E-01	N
Tox2	TOX high mobility group box family member 2	8.6E-03	1.7E-01	N
Sorcs1	VPS10 domain receptor protein SORCS 1	8.5E-03	1.7E-01	N
Slc6a1	solute carrier family 6 (neurotransmitter transporter, GABA), member 1	8.5E-03	1.7E-01	N
Mal2	mal, T-cell differentiation protein 2	8.5E-03	1.7E-01	N
Mgat4c	mannosyl (alpha-1,3-)-glycoprotein beta-1,4-N-acetylglucosaminyltransferase, isozyme C	8.5E-03	1.7E-01	N
Bdh1	3-hydroxybutyrate dehydrogenase, type 1	8.0E-03	1.7E-01	N
Pter	phosphotriesterase related	8.0E-03	1.7E-01	N
Lrp4	low density lipoprotein receptor-related protein 4	7.6E-03	1.6E-01	N
Hcn1	hyperpolarization-activated, cyclic nucleotide-gated K+ 1	7.5E-03	1.6E-01	N
Ctsf	cathepsin F	7.5E-03	1.6E-01	N
Nts	neurotensin	7.2E-03	1.6E-01	N
Rgs16	regulator of G-protein signaling 16	7.0E-03	1.6E-01	N
Ndufa4l2	NADH dehydrogenase (ubiquinone) 1 alpha subcomplex, 4-like 2	7.0E-03	1.6E-01	N
Eml3	echinoderm microtubule associated protein like 3	6.9E-03	1.6E-01	N
Hopx	HOP homeobox	6.7E-03	1.6E-01	N
Nr2e3	nuclear receptor subfamily 2, group E, member 3	6.6E-03	1.6E-01	N

Cacna2d4	calcium channel, voltage-dependent, alpha 2/delta subunit 4	6.6E-03	1.6E-01	N
Vsx2	visual system homeobox 2	6.5E-03	1.6E-01	N
Zranb1	zinc finger, RAN-binding domain containing 1	6.5E-03	1.6E-01	N
Hkdc1	hexokinase domain containing 1	6.0E-03	1.5E-01	N
Slc27a2	solute carrier family 27 (fatty acid transporter), member 2	6.0E-03	1.5E-01	N
Npr3	natriuretic peptide receptor 3	5.6E-03	1.5E-01	N
Prkg1	protein kinase, cGMP-dependent, type I	5.3E-03	1.5E-01	N
Cdh11	cadherin 11	5.2E-03	1.5E-01	Y
Samd7	sterile alpha motif domain containing 7	4.8E-03	1.4E-01	N
Dpp10	dipeptidylpeptidase 10	4.8E-03	1.4E-01	N
Crim1	cysteine rich transmembrane BMP regulator 1 (chordin like)	4.7E-03	1.4E-01	N
Cngb1	cyclic nucleotide gated channel beta 1	4.3E-03	1.3E-01	N
Pias3	protein inhibitor of activated STAT 3	4.0E-03	1.3E-01	N
Rbl1	retinoblastoma-like 1 (p107)	3.9E-03	1.3E-01	N
Rab9b	RAB9B, member RAS oncogene family	3.6E-03	1.2E-01	N
Ttyh2	tweety homolog 2 (Drosophila)	3.5E-03	1.2E-01	N
Slitrk4	SLIT and NTRK-like family, member 4	3.4E-03	1.2E-01	N
Unc5d	unc-5 homolog D (C. elegans)	3.3E-03	1.2E-01	N
Cnga1	cyclic nucleotide gated channel alpha 1	3.1E-03	1.1E-01	N
Smpdl3a	sphingomyelin phosphodiesterase, acid-like 3A	3.0E-03	1.1E-01	N
Ppic	peptidylprolyl isomerase C	3.0E-03	1.1E-01	N
Sema3e	sema domain, immunoglobulin domain (Ig), short basic domain, secreted, (semaphorin) 3E	2.9E-03	1.1E-01	N
Kctd8	potassium channel tetramerisation domain containing 8	2.7E-03	1.1E-01	N
Glt25d2	glycosyltransferase 25 domain containing 2	2.7E-03	1.1E-01	N
Klhl1	kelch-like 1 (Drosophila)	2.2E-03	1.0E-01	N
Slc1a6	solute carrier family 1 (high affinity aspartate/glutamate transporter), member 6	1.8E-03	8.9E-02	N
Tmem55a	transmembrane protein 55A	1.2E-03	6.7E-02	N
Zic1	zinc finger protein of the cerebellum 1	1.2E-03	6.7E-02	N
Pak3	p21 protein (Cdc42/Rac)-activated kinase 3	1.1E-03	6.6E-02	N
Tmem47	transmembrane protein 47	1.0E-03	6.3E-02	N
Plagl1	pleiomorphic adenoma gene-like 1	9.9E-04	6.3E-02	N
9230110C19Rik	RIKEN cDNA 9230110C19 gene	8.7E-04	5.9E-02	N
AI593442	expressed sequence AI593442	6.8E-04	5.2E-02	N
Lhx9	LIM homeobox protein 9	5.3E-04	4.7E-02	N
Col9a1	collagen, type IX, alpha 1	5.1E-04	4.6E-02	N
Tpd52	tumor protein D52	5.0E-04	4.6E-02	N
Fgd4	FYVE, RhoGEF and PH domain containing 4	4.9E-04	4.6E-02	N
Prom1	prominin 1	4.0E-04	4.1E-02	N
Slc1a3	solute carrier family 1 (glial high affinity glutamate transporter), member 3	3.5E-04	3.8E-02	N
Chrnb4	cholinergic receptor, nicotinic, beta polypeptide 4	3.0E-04	3.7E-02	N
Islr2	immunoglobulin superfamily containing leucine-rich repeat 2	2.7E-04	3.5E-02	N
Tulp1	tubby like protein 1	1.8E-04	2.8E-02	N
Fgf14	fibroblast growth factor 14	1.5E-04	2.4E-02	N
Slc12a6	solute carrier family 12, member 6	1.2E-04	2.1E-02	N
Gad1	glutamic acid decarboxylase 1	7.8E-05	1.6E-02	N
Tnfaip6	tumor necrosis factor alpha induced protein 6	7.6E-05	1.6E-02	N
Zic3	zinc finger protein of the cerebellum 3	6.8E-05	1.6E-02	N
Bin1	bridging integrator 1	5.4E-05	1.5E-02	N
Rom1	rod outer segment membrane protein 1	3.8E-05	1.2E-02	N
Ppef2	protein phosphatase, EF hand calcium-binding domain 2	2.6E-05	1.1E-02	N
Kdr	kinase insert domain protein receptor	2.4E-05	1.1E-02	Y
Prdm1	PR domain containing 1, with ZNF domain	2.4E-05	1.1E-02	Y
Nrl	neural retina leucine zipper gene	2.2E-05	1.1E-02	N
Epb4.1l2	erythrocyte protein band 4.1-like 2	1.8E-05	1.1E-02	N
Epha5	Eph receptor A5	1.0E-06	3.3E-03	N

red shading indicates genes that are upregulated in expression and the epigenetic marks match that change
green shading indicates genes that are downregulated in expression and the epigenetic marks match that change
cancer gene designation is taken from the Sanger Cancer Gene List
Empirical p value was caludated by permutation analysis
FDR adjusted empirical p value was calculated using Benjamini-Hochberg method. Only genes with FDR <0.25 are shown in this table

Supplemental Table 11: Exome sequencing validation primer sequences

Gene	Chr	Position	SNV/Indel	Forward	Reverse
Bicd1	chr6	149460634	A/C	CATATCCATGGGCCTCTTGGGAAAC	TGTTGAATTTGGGGACTTCTGTGTTCTT
Napb	chr2	148523913	A/G	AGATGGCACTGATCCTCTGCAACTG	TGAAGTATGCAGCCTTGAAATCGCA
Lpcat2	chr8	95441991	A/G	TGTATTCACGGCTCCTGTTGGTCTG	CCCTCCTCCTGGCTCTCAGGACT
Cdk7	chr13	101481476	A/G	TAACTCAGTGACGCTCCTTCCTCGG	ATGTGGGTGCTCCCGTTTACCAAAT
Kdm4c	chr4	73944424	G/T	TCCCTGTCTTGTCTCTAGGGTGTGGA	CCCTGGCTGTCCTGGACCTTACTCT
Dync2h1	chr9	7102432	C/T	TCAGTTGCCCACAACCTTGATATCCT	TGGGAACAGAACCTAGGTCCAGCAA
Wdr20a	chr12	112032438	G/T	TCGCTACATGACCGGAAGGAGAGAC	ACCCAGAGGGATGATTGACTTGGCT
Zfp493	chr13	67887813	g/A	GTGCTCGCACAACGCTCTCTAAACA	ATGGATTCGCAGATGCTTATGCAGG
Olfr1030	chr2	85824701	C/T	CCATTTCATATGCTGCGTGTTTAGTCCA	TCTAACATACATGCAGAATAGCGTCCCA
Gm6760	chrX	61404728	C/A	CAGCCTGCTGGAGATGTTGCAGAG	AATGAAGGAGAAGGGCCTGTTCAGC
Crp	chr1	174628313	C/T	TGGAGCCTTGGGAAGCTTTCTCATT	ATCAAGGGTCATCTGGTCCTCTCCC
Slc43a3	chr2	84797981	C/G	CATTGGTCCATCCCTTCCTGGTGTA	AGCCAGTCAGTTGGATCCCAAGTCC
D630045J12Rik	chr6	38141752	C/T	GGCTCAGGGTCTCACCTTTAGCTCC	CCCAACAGCTGCTTTGAACCTCAGT
Gpr135	chr12	73170742	C/G	GCCGCATTTGAAGAAAGGGAATGTT	CAACCGCGAAGAAGGGTACAGGACT
Arhgap17	chr7	130465173	C/T	GCTTCTCTCTGCTCCCTTTCCTTGG	GGAGATCATGGACCCTCTCTACGGC
Ftsjd2	chr17	29828357	G/A	GCCTCCCAAGGAGGATTTAAGGCAT	GACTCCTTGGAACAGGACCAGCAGA
Spin2	chrX	150267969	C/A	GGAGAAGGCGTAGGTCACCACATTG	GAGGAGATGCACTGGACTCTGGCAT
Ttll4	chr1	74734779	A/G	TTTGCCAGTTGCAAGTATGTGCTGG	TTCCTTCCCAGGTTCAAGACGAACA
Naip2	chr13	100914505	T/C	TCTTAAGCACTGGGCCATCTCTCCA	GTTGCTTTCTTTCCAGCCAAGGGAG
Afg3l1	chr8	126004266	C/A	GTTCTGTCCCTTGCATCCTGTCCTG	CTGTGCTCCAGGTAAACTGGGCTGT
Ceacam2	chr7	26323764	C/T	CTTCATGGTGACCCCTTTGGATGAGC	ATGAAGGCGCAGGGTCTTTAGAGGA
Mga	chr2	119790348	G/T	GGACACTTGGGAGCAGGTGTGAAAG	AACAGGTGCCAATGTAGGCATCACC
C030046E11Rik	chr19	29677094	C/T	GATGGATCCCTCTGCGTACTTGCTG	CTGGATGACGGGAAGCCTGCTATTT
Zfp618	chr4	62757967	G/T	CCTGTGGGTTGGGAAGACAATGAAA	GAGATCCGCCTGTCTTTGCTGTCTG
Tatdn2	chr6	113654793	G/T	CCACAGAGAGCTGAGCCTGGAAGAG	ATTCAAGGACAGCCAGGGCTACACA
Slc25a15	chr8	23488450	A/G	GACCAGTCACTAAACGCCTGCCTTG	GTTCAGTCTCCCTCATGACCACGCT
Il20	chr1	132807865	T/C	ACACAGCTTCCCAAATGGAGGGTCT	GCAGAAGGAGTTGCAGTGGCTTTCT
Tram2	chr1	21003482	C/T	CTGTGGTTTCCTCCAAGGAAGAGGG	TCACAGTGCCATGGGAAGTAAAGGG
Smpd4	chr16	17640315	T/C	GCTACCTGTCTTCAGGGCACCCTTC	GTGCTAGCAGAGTGACAGCAGCCAA
Slc10a4	chr5	73403214	G/C	TCCTTCCATCCCAAGCAAGTGAAGA	ACTGGAAGAGGGCGTAGAGCAGAGG
Lig3	chr11	82610799	C/A	CCTTGAACTCAGAAATCTGCCTGCC	CTTCCCTCTTCAGAGAGGCTGTGGC
Purg	chr8	34497428	G/T	AACTGACCCTCTCCCTGTCGGTAGC	GTCGTCGTCTCCACACCTTCGTTCT
Zfp128	chr7	13476431	G/A	CTGTGGGAGGGCCTTCAATCAGAAT	GGTTCTTCCTCTCTGCTGTGGGTGA
4930590J08Rik	chr6	91869226	C/T	CTCTCTGGAGAGTGAGAACCGAGCG	AGAGCCTGACCTGGACCAACACTTG
Rgs3	chr4	62362073	T/A	GTTGGGACCTGTGTGCTCAGACCAT	TGACCTTCTTGAAGTCCTCGCAAGC
Bap1	chr14	32065142	T/C	GCACCTGCATGACAAATCCCTCTTC	GTGCAACACTATGCAACGGAGGGTT
Afap1	chr5	36278681	C/A	TCCAGATTCCCAGGGTTCCAAATTC	AGGAACGTGAGTAGCCCAGACCTCC
Epn2	chr11	61359740	G/A	CAATCTAGATCGAGGACGCTGTCGC	GCGGCATGTATACAAGGCACTGACA
Nos1	chr5	118373362	A/G	TCCTGGACAGGTTCTCAGTCTTCGG	GGCAGTGAATGGGACAGAGAGCAAA
Nos1	chr5	118317770	C/A	AGGTCCCAGTAACGGACCTCAGCAT	TCCCTGGACCGTGGTTGTTAGTGAC
Pkd1	chr17	24708577	C/A	TGGTAGAGGCTGGCTCAGACGTAGC	TAGGCCCAGAAGCACTCACAGGAAG
Gtse1	chr15	85692913	T/G	TTGCTGCCAGTTTGGACTTAAACCG	ACTTGATTCTCTTGGCAACGGCTGA
Card11	chr5	141354018	C/T	GCTCACCTGACTTGCAGATGGTGAA	TGGCTGTACTCCTTAGACCCTGCCC
Gripap1	chrX	7389985	T/C	AGCAACCTGAAGCAGGTCTGTGGTC	AGCAAGGCTCAGGGAGGAATGAAAG
Gse1	chr8	123086637	G/T	CACCGGAGGTACATTGACGTGGTTT	GGCTCTCGCTTCTCCAGACTCCATT
Stx5a	chr19	8816850	A/G	CAGTCAGAGCCAAGTCGGAAGGAGA	TAACTAAATGGGCTCACGCCTGCCT
Dnahc2	chr11	69291587	G/A	CTCGAGTTCCAGCCTCTTTCAGCAC	AGATGCACTTTCCATCCTGGGTCCT
Zfp213	chr17	23697057	C/A	ATCCAACACCTCTCCTGTCTGTCGC	AAGGGCTGTGTCCCAGAACTCCTTC
Irx2	chr13	72768205	G/A	GTACCGTAAGAACGCTACGCGGGAC	TAAATGCGGCCCAGCATCTCTACA
Abhd16a	chr17	35233465	C/G	GGTGGATGGCTAGGACTTCCCTCAG	ACAGCACTAGGCCCTACAAGCGACA
Fam100a	chr16	4875876	G/T	CTTGGTTCTGAGGTGGCCTGTTGAG	CCTGAAGCCAGGCTAACCCTTTGTC
Chst5	chr8	114414209	C/A	ATCCCTGGATGTGGTCTTGAAGGCT	GCCTGCAGCTCCTATAGTCACGTCG
2410016O06Rik	chr12	85292543	C/A	ACTACGAGGGTCTCTTCTCCACCGC	CTCCAGGTTCCAGTACCGTTTGCAG
Gpr123	chr7	147033644	G/-	CTTTCTCATGGTCTGCTCCTTCGGA	GGCCAAACAGCCTGCTTCAGATG
Heatr7b1	chr1	90128834	C/-	ACATCAGCCTTCTCCCTGTCCTGTG	CAACCACTGGGATGCAAACCAACTT
Tmco7	chr8	109266076	A/-	AATAGCTCAGGGCAGTGCTGAGTGC	CCCGGAACTGTTGTGTGGTTATGCT
Gde1	chr7	125842064	C/A	TGGACACACAGTGCTTCTGTTGCTG	AAGCTTGAGGCAGCATTAACACCCA
Ppp2r5a	chr1	193178493	A/-	AGACCATTTGCTGAGGAGCATCCAT	CCAAGTGCGTCTCCAGCTCTCATTT
Ly9	chr1	173527817	T/-	GGTACCAAGTGCACACGTGATGTCTGT	AACCTCTTGCCTAGCAGGCTTGGTG
Cpsf4	chr5	145942096	T/AGCAGC	TGTGGTGAGAAAGGACACTACGCCA	AGGTGCTGGTGTTTAAATGAGGCCC
Treml2	chr17	48442063	A/-	TCCGGCTATGTTCTTGCCTCACACT	CACTTCCAGTTGGAAACCCACCAAA
Bicc1	chr10	70394957	C/G	GCTGGAAGACATCCGTGTATTTGCC	AGCCTGGACCTGTGGTCACTTCTTG

Supplemental Table 12: Histone mark scoring system

Combined score	H3K4me3	H3K9/14ac	H3K9me3
1	1	1	-1
2	1	1	0
2	1	0	-1
2	0	1	-1
3	1	1	1
3	1	0	0
3	0	1	0
3	0	0	-1
3	-1	1	-1
3	1	-1	-1
4	1	0	1
4	1	-1	0
4	0	1	1
4	0	0	0
4	0	-1	-1
4	-1	1	0
4	-1	0	-1
5	1	-1	1
5	0	0	1
5	0	-1	0
5	-1	-1	-1
5	-1	1	1
5	-1	0	0
6	0	-1	1
6	-1	0	1
6	-1	-1	0
7	-1	-1	1
